# Size and Shape Modulation of Cu_2_S Nanoplates via Chemical Reduction with NaOH and NH_3_·H_2_O

**DOI:** 10.1021/acsomega.4c06316

**Published:** 2024-10-30

**Authors:** Vinh-Dien Le, Gabrielle J. Grey, Ill-hyuk Han, Mark D. Hammig

**Affiliations:** † College of Engineering, University of Michigan, Ann Arbor, Michigan 48109, United States; ‡ Amphionic LLC, Plymouth, Michigan 48170, United States; § Department of Nuclear Engineering, Seoul National University, Seoul, South Korea, 08826

## Abstract

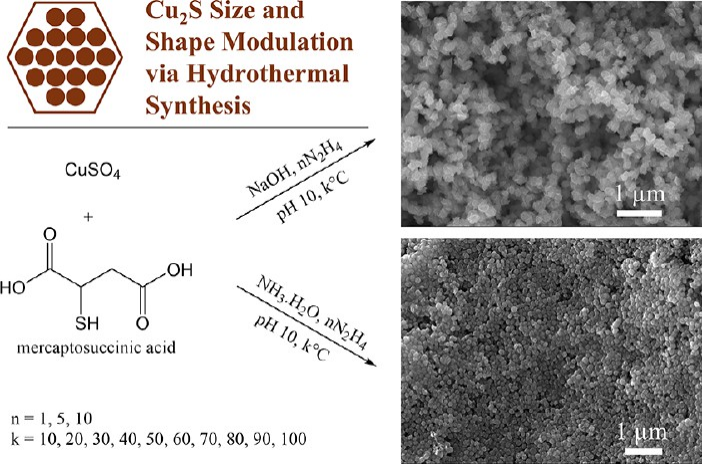

Copper(I) sulfide (Cu_2_S) has electrical, optical, and thermoelectric properties that make it a promising material for a variety of applications, including energy conversion and antibacterial coatings. Nevertheless, the current synthesis and morphological modulation of Cu_2_S typically focuses on thermolysis of the copper and sulfur precursors, is procedurally complex, and demands expensive equipment. In this article, a facile, high-yield, three-step, low-temperature aqueous synthesis alternative for Cu_2_S nanoplates is introduced. By variations of the reaction temperature, reducing agent concentration, and pH modifier (NaOH or NH_3_·H_2_O), the morphological characteristics can be controlled. As confirmed with scanning and transmission electron microscopy, the lateral extent of the synthesized Cu_2_S nanoplates can be tuned from around 30 nm to around 300 nm simply by varying the heating conditions from 10–100 °C. A similar effect is more subtly observed by varying the concentration of the reducing agent. In addition to size variance, the morphological properties of the Cu_2_S nanoplates can be changed by using different bases for the reaction. Characterization of the composition and crystalline structure of the materials has also been performed using energy-dispersive spectroscopy and X-ray diffraction, and optical properties are investigated by UV–visible and near-infrared spectroscopy (UV–vis–NIR). The synthesis pathway described in this paper can be easily performed and feasibly scaled, which is advantageous as the retrieved material is suitable for diverse applications, such as its use in battery electrodes, photonic and charged-particle sensors, and radiation shielding.

## 1 Introduction

Cu_2_S has been studied intensively for multiple energy applications due to its physical properties that are suitable for the manufacture of photovoltaic devices and catalysts. As a semiconductor with a bulk band gap of 1.2 eV,^1−3^ different Cu_2_S nanospecies (plates, rods, flowers, etc.) are studied for potential applications in different fields such as battery electrodes,^4−6^ solar cells,^7,8^ and catalysts.^9−11^ In these applications, the size and structure of the nanomaterial greatly affect the performance of each application, motivating the desire to gain control over these properties. For battery electrodes, Deng et al. 2020^4^ synthesized Cu_2_S nanoplate electrodes with a specific surface area of 6.2 m^2^ g^–1^ that delivers a good specific capacity of 206.6 mA h g^–1^ at 2000 mA g^–1^ after 400 cycles for potassium-ion batteries. For solar cells, Wu et al. 2008^8^ synthesized colloidal Cu_2_S 5 nm hexagonal nanoplates incorporated with CdS to make a solar cell that produces open-circuit voltage of 0.6 V and short-circuit density of 5.63 mA/cm^2^.

Up to this point, most of the synthesis pathways for Cu_2_S nanomaterials involve a top-down approach that uses high temperatures to form the compound. Conventionally, the most common way to synthesize Cu_2_S is to perform thermolysis on organometallic compounds that contain both Cu and S,^4,12−14^ whereas other methods to synthesize Cu_2_S may include the use of microwave radiation.^15^ Specifically, in 2020, Deng et al. reported a Cu_2_S nanoplate synthesis pathway for anode fabrication by baking a mixture of copper nitrate and citric acid at 160 °C before grinding the mixture with sulfur powder inside a furnace at 400 °C for 2 h.^4^ In another article, Wang et al. 2000,^14^ reported a one-pot synthesis of Cu_2_S nanoplates by the hydrothermal reaction of cuprous acetate, tri-*n*-octylphosphine oxide, and 1-octadecene. The reaction needs to be carried out under argon flow and heating from 160 °C and above to achieve size modulation. A solventless synthesis pathway was also introduced by Sigman et al. 2003^16^ where copper nitrate, chloroform, sodium octanoate, and dodecanethiol are reacted together to form a two phase system where the aqueous phase is discarded after 10 min, and the organic phase is heated from 140 °C and above to obtain solid Cu_2_S nanodisks. Regarding the microwave-assisted synthesis technique, Mousavi-Kamazani et al. 2013^15^ first made bis(salicylate)copper(II) from copper nitrate and sodium salicylate via a hydrothermal reaction after which bis(salicylate)copper(II) is dissolved in propylene glycol with the further addition of sodium sulfide, hydrochloric acid, and thiosemicarbazide. The mixture was then loaded into a Teflon container to perform microwave digestion. All of these methods by which Cu_2_S nanostructures are synthesized require heating to above 100 °C at some stage, and most of the methods require the use of multiple reagents, especially multiple organic compounds, and many of the reactions require an inert environment. For the most involved reactions, such as that described in the paper reported by Sigman et al. 2003, all of the lengthy reaction steps eventually lead to a typical yield of only 10–20%.^16^

In this paper, we report a facile three-step, one-pot, high-yield synthesis of Cu_2_S nanoplates with only four reagents inspired by previously reported chemical reduction mechanism used for the synthesis of metallic nanoparticles.^17^ The synthesis is conducted at low temperature (under 100 °C) and under ambient conditions without the need for an inert environment. By variation of temperature, reducing agent concentration, and base addition, this synthesis pathway results in nanoplates that are tunable in terms of their structure and size which can be explained by examining the LaMer’s burst nucleation mechanism for nanoparticle synthesis as well as particle surface chemistry. The results reported below also show that the prepared Cu_2_S nanoplates can integrate into porous polymeric matrices, such as a hydrogel formed from aramid nanofibers (ANFs) which means that the prepared material is capable of being incorporated into a free-standing composite solid. The prepared colloidal solution can therefore be readily integrated into flexible composites and can thus serve as a potential candidate for electrodes, lightweight radiation sensors, or shields. These further outlooks and implications are also discussed in this article. In Table 1, a summary table of several studies on Cu_2_S nanoplates synthesis compare to this article route is made to help compare current Cu_2_S nanoplates syntheses with this paper’s synthesis.

**Table 1:** Summary Table for the Previous Synthesis Routes of Cu_2_S Nanoplates

source	method	temperature	environment	time	size	yield
Sigman et al. 2003^16^	solventless thermolysis	≥140 °C	ambient	>40 min	3–150 nm (diameter) 3–12 nm (thickness)	10–20%
Chen et al. 2008^12^	thermolysis	200–220 °C	N_2_	>10 h	3–27.5 nm (diameter) ∼12 nm (thickness)	NA
Wang et al. 2010^14^	one-pot thermolysis	≥160 °C	Ar	>1 h	<3–20 nm (diameter) ∼10–∼20 nm (thickness)	NA
Mousavi-Kamazani et al. 2013^15^	chemical reduction assisted with cyclic microwave radiation	microwave power ≥300 W	microwave	>10 h	∼9 nm (crystallite diameter)	NA
An et al. 2015^9^	phase transformation from CuS	220 °C	N_2_	>1 h	15.9 nm (diameter)	NA
Deng et al. 2020^4^	solventless	400 °C	Ar	>2 h	NA	NA
**this work**	aqueous chemical reduction	**≤100 °C**	**ambient**	<1.5 h	∼30–∼300 nm (diameter) ∼5–∼20 nm (thickness)	**39.25–77%**^a^

a Only two samples were used to calculate the yields for this work.

## 2 Experimental Details

### 2.1 Reagents

Copper(II) sulfate (CuSO_4_), anhydrous powder, ≥99.99% trace metal basis, Sigma-Aldrich; mercaptosuccinic acid (C_4_H_6_O_4_S—MSA), for synthesis, ≥98.0%, Merck; ammonium hydroxide solution (NH_3_ in H_2_O or NH_3_·H_2_O), puriss. p.a. plus, ≥25%, Sigma-Aldrich; sodium hydroxide (NaOH), pellets, ≥97.0%, ACS reagent; hydrazine hydrate solution (N_2_H_4_.H_2_O), puriss. p.a., 24–26% in H_2_O (RT), Sigma-Aldrich; potassium hydroxide (KOH), pellets, ≥85%, Sigma-Aldrich; triethylamine ((C_2_H_5_)_3_N—Et_3_N), ≥99.5%, Sigma-Aldrich; dimethyl sulfoxide (C_2_H_6_OS—DMSO), ACS reagent, ≥99.9%, Sigma-Aldrich; Kevlar threads, DuPont; and deionized water were used.

### 2.2 Cu_2_S Nanoplates Synthesis

**Figure 1:**
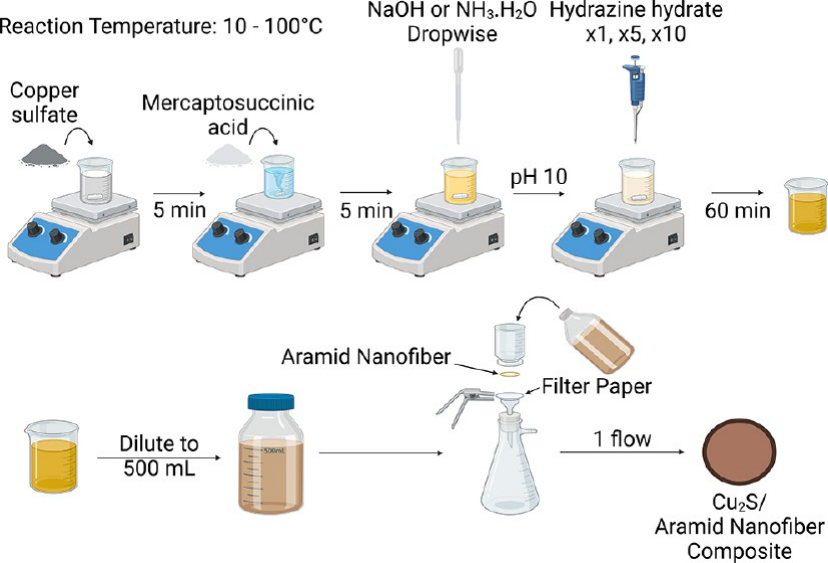
Experimental scheme made with BioRender.^18^

Cu_2_S nanoplates are prepared in aqueous media and under ambient condition via a three-steps synthesis pathway that is illustrated in Figure 1. 100 mL portion of deionized water is first added to a 250 mL three-neck round-bottom flask with a 3.5 cm oval stir bar. The round-bottom flask is set on a heating mantle where the reaction temperature can be controlled from 10 to 100 °C with a margin of error within ±2 °C, and the rotation speed is set to 500 rpm. In the flask, 0.08 g (0.5 mmol) of CuSO_4_ is then dissolved and rigorously stirred for 5 min. Subsequently, 1.5 g (10 mmol) of MSA is added to the flask and reacted for an additional 5 min. The solution, after briefly (3 s) turning purple with the addition of MSA, becomes a light, translucent yellow. This phenomenon can be explained by an oxidation–reduction interaction between Cu and thiol compound.

Specifically, when excess thiol is added to the copper solution, a transient product Cu^II^—(RS)_2_ is formed.^19^ This transient product is then quickly reduced into a Cu^I^ species, and disulfides bonds are formed as the auto-oxidation of thiols occurs.^19,20^ When synthesis occurs under ambient conditions, the Cu^I^ species will also undergo oxidation with O_2_ to reform Cu^II^.^19,20^ This rapid oxidation of Cu^I^ can be prevented by incorporating excess thiol into the reaction. This is accomplished by using 20 times MSA relative Cu on a molar basis. As MSA is an acid, its addition will bring the pH down to around 2. If heat is introduced in the reaction, the pH of the solution after this step can get as low as 0.5.

A 3 M NaOH solution or 25% NH_3_·H_2_O solution is then added dropwise to the solution to alkalize it to a pH of 10. It is observed that with each dropwise addition of the base, the color of the solution darkens until the solution reaches a pH of around 7–8 where the solution suddenly turns translucent. It is hypothesized that this phenomenon is caused by the full deprotonation of all carboxylic acid and thiol sites of MSA as the p*K*_a_ values of these functional groups are typically around or under this pH range. The solution’s pH change is also more sensitive at this range until it reaches 9 where it increases at a slower rate up to 10, indicating that the solution is getting to a buffer state. Note that for samples that run with NH_3_·H_2_O at elevated temperature, it could take up to around 60 mL of the base to reach pH 10. From a pH of about 8–10, the solution will darken again with each base addition. This change can be attributed to interactions between the base and the copper ions, including complexation with the hydroxide group. Finally, different N_2_H_4_ concentrations ranging from 0.064 mL (0.5 mmol) to 0.32 mL (2.5 mmol) or 0.64 mL (5 mmol) were added at once in the solution. The flask is then sealed with rubber stoppers and left to react for 60 min.

When the reaction is carried out at or above 80 °C, a reflux condenser is attached. The solution typically remains a dark brown color or light black. It is also observed that color change of the solution after addition of N_2_H_4_ happens much faster for samples that are made with NaOH, occurring within several minutes, compared to the color change of samples that are made with NH_3_·H_2_O, which can take the whole 60 min period to appear significantly darker. Furthermore, the solution will change color faster at this stage if heating is introduced. These phenomena are further discussed below. After the reaction is completed, the solution is quenched by dilution to 500 mL (5×) using cold deionized water. In the case of precipitation at the bottom of the flask after the reaction is completed, dilution also helps redisperse the particles within the colloid. The diluted solution is then integrated, via vacuum filtration, into an ANF hydrogel, which acts as a scaffold for the nanoplates.

### 2.3 Aramid Nanofiber Synthesis

ANF is prepared by a modified procedure introduced originally from Yang et al. 2011.^21^ Specifically, 2% ANF is made by dissolving 4 g of Kevlar thread and 4 g of ground KOH pellets in 180 mL of DMSO. The solution is stirred for 2 weeks to ensure that the Kevlar is fully dissolved. The resulting thick, red, viscous dispersion is spun-cast (300 rpm, 5 s spread, and 15 s spin) upon a 5 cm-diameter circular glass slide and immediately submerged in deionized water to remove the DMSO and protonate the ANF. The thin film is hydrolyzed when its color is an opaque white, which typically takes around 10 min. For references, Figure S1 in the Supporting Information sections shows additional scanning electron microscopy (SEM) images of the ANF while Figure S35 shows the X-ray diffraction (XRD) graph of the ANF.

### 2.4 Characterization

Characterization of the Cu_2_S nanoplates have been performed using SEM and transmission electron microscopy (TEM), energy-dispersive spectroscopy (EDS), and XRD. SEM images with EDS spectra are collected with a JSM-IT500HR InTouchScope scanning electron microscope which has a resolution of 1.5 nm (0.5–30 kV). TEM images with EDS spectra are collected with a Thermo Fisher Talos F200X G2 S/TEM which has a resolution of below 0.16 nm for STEM mode (80 and 200 kV). XRD spectra are taken using the Rigaku SmartLab X-ray diffractometer (Cu Kα, 40 kV, 50 mA, step 0.01°, 5.0°/min, incident slit 2/3°, receiving slit 20.000 mm). Optical measurements of the colloids are collected using a JASCO V-770 UV–vis–NIR spectrophotometer (Czerny-Turner mount, single monochromator, double beam). To measure the average diameter of the nanoplates, typically, 50–100 of clearly visible nanoplates are randomly sampled at 10–20 randomly selected fields on the SEM views. This method has been proven to be relatively reliable when compare to size measuring using a dynamic light scattering device zetasizer Nano ZSP (range ∼0.3 nm to 10 μm). For reference, Figure S2 in the Supporting Information section shows the size distribution measurements taken using a dynamic light scattering device zetasizer Nano ZSP for Cu_2_S samples synthesized with 0.064 mL of N_2_H_4_ (1×) at 20 °C using NH_3_·H_2_O.

### 2.5 Gamma-ray Interaction Simulations

The thickness of the solid was measured via calipers (7 ± 1 μm). The simulated thickness was 20 times this value to account for the enhanced electron stopping that accompanies nanostructuring the solid.^22^ The lateral area of the Cu_2_S/ANF solids (1.7 cm^3^) were measured via calipers, and the density (1.47 g/cm^3^) was derived from the measured volumes and the measured mass (0.0088 g). The relative weight fractions of the inorganic (Cu, S) and organic (C, N, O, S) constituents were derived from EDS, as shown in the Supporting Information section. The Monte Carlo N-particle (MCNP6^23^) code simulation included the target, an assumed point ^133^Ba source 1 cm distant from the source and the aluminum block upon which the sensor was placed in order to simulate the effects of aluminum backscatter. The ^133^Ba energy distribution consisted of the following peaks (in MeV): 0.00467, 0.03063, 0.03097, 0.03492, 0.03499, 0.03525, 0.035822, 0.035907, 0.035972, 0.053, 0.0796, 0.0809, 0.1606, 0.2232, 0.2763, 0.3028, 0.3560, and 0.3838, with the following relative intensities: 0.03725, 0.07934, 0.1464, 0.01427, 0.01427, 0.01427, 0.00347, 0.00347, 0.00347, 0.00502, 0.0263, 0.3331, 0.00638, 0.0045, 0.0713, 0.1831, 0.6205, and 0.0894.^24^ In general, a total number of 10^9^ photons was simulated per run, and the F8 (energy deposition) tally was used to capture the spectrum. The subsequent charge electron–hole transport within the sensor was not included as we carried out in 2019^25^ because of negligible charge trapping within the sensor, as evinced via the sharp rise (<1 μs) time and symmetric energy peaks.

### 2.6 Gamma-ray Interaction Measurements

A 31.55 MBq ^133^Ba X-ray and gamma-ray disk source (radius of 5 mm) was placed upon a polypropylene stand and separated from the sensor by 2 cm. Both source and detector were placed within a vacuum chamber test box that was evacuated during measurements. The sensor sat upon an aluminum test stand (ground contact) and was contacted on its top surface via a copper wire. The gamma-ray spectra were collected with an Ortec 142A charge sensitive preamp, an Ortec 572A shaping amplifier (shaping time: 500 ns), and digitizer (CAEN DT5730B). The sample was biased to 80 V, and the spectrum was collected for 1000 s.

## 3 Results and Discussion

### 3.1 Effect of the Temperature

**Figure 2:**
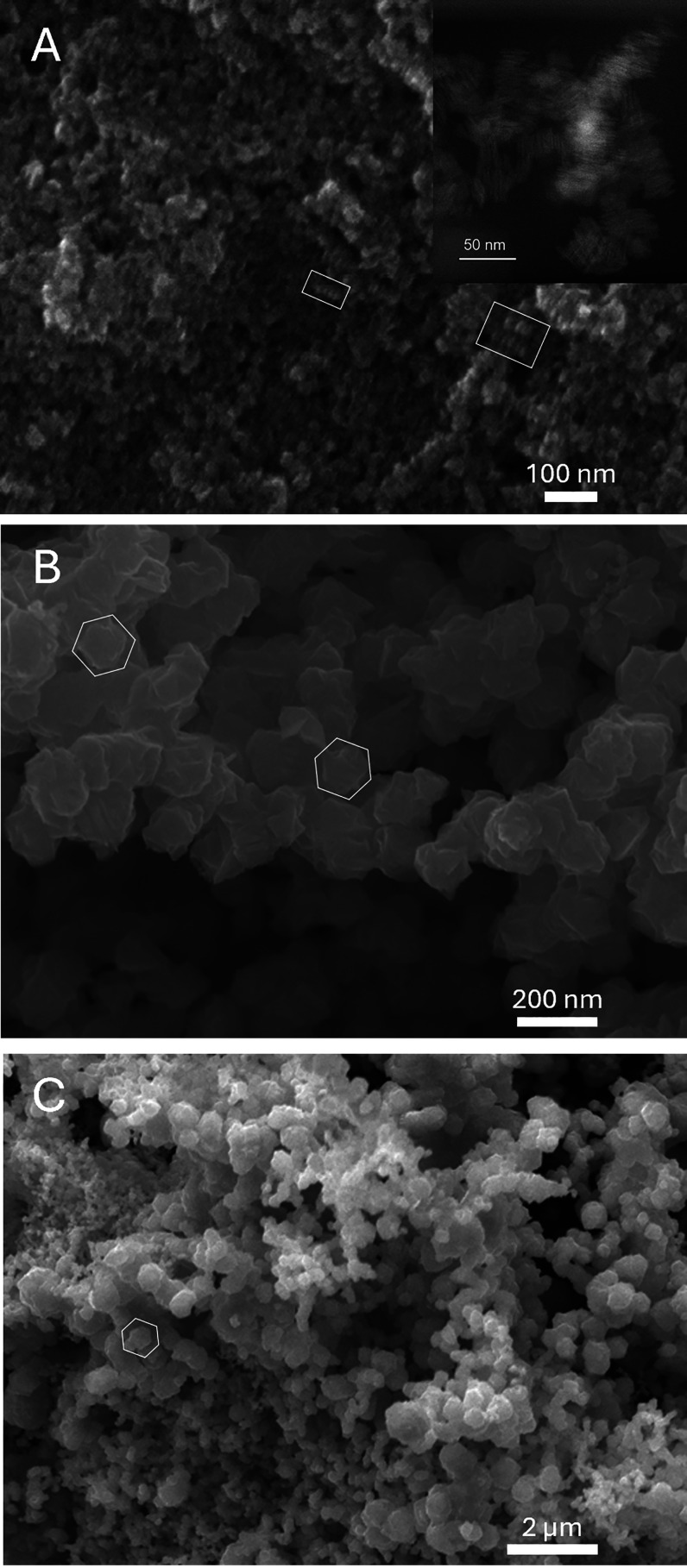
SEM images of Cu_2_S samples synthesized with 0.064 mL of N_2_H_4_ using NaOH as the base at (A) room temperature (20 °C) with a TEM image on the inset taken to better visualize the nanoplates; (B) 40; and (C) 80 °C; MOE = ±2 °C. White hexagons indicate the shape of the plates, whereas white rectangles indicate the self-assembly of the plates.

**Figure 3:**
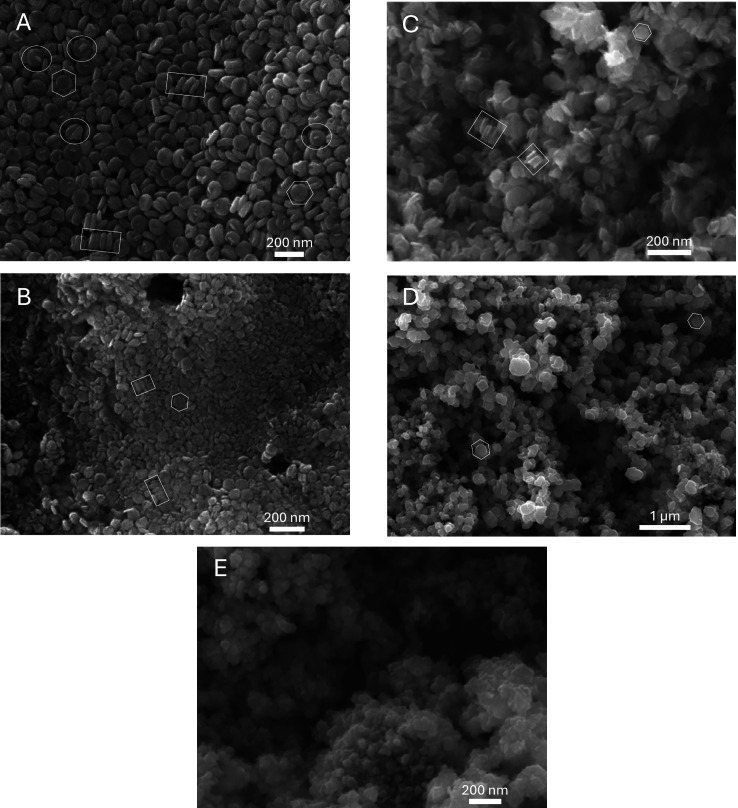
SEM images of Cu_2_S samples synthesized with 0.064 mL of N_2_H_4_ using NH_3_·H_2_O as the base at (A) room temperature (20 °C); (B) 40; (C) 60; (D) 80 °C; and (E) 100 °C MOE = ± 2 °C. White hexagons indicate the shape of the plates, white rectangles indicate the self-assembly of the plates, and white ovals indicate the twinned plates.

For size-modulation via temperature variation, the reaction time (60 min) and the amount of reducing agent used are kept constant at 0.064 mL of N_2_H_4_, while the temperature is varied from 10 to 100 °C. The resulting sizes and structures from this modulation are shown in Figures 2 and 3, for NaOH and NH_3_·H_2_O, respectively. The overall trend in the size modulation is plotted in Figure 5.

The variation of the reaction temperature for Cu_2_S nanoplates synthesis results in different size and shape trends for the cases in which the base is varied, using either NaOH and NH_3_·H_2_O. As shown in Figures 2 and 3, both synthesis pathways produce plate-like Cu_2_S nanoparticles with high aspect ratios. Although the crystallites formed via the NaOH route have relatively high size dispersion and abnormal shapes which is likely due to aggregation, some hexagonal structure of the nanoparticles is highlighted in the white hexagonal outlines in Figure 2B,C, a structural form consistent with that of high chalcocites as reported in multiple studies.^12−14,16^ On the other hand, the aggregation of the nanoplates in Figure 2 is likely due to the fact that the surface energy of these nanoplates is higher due to their relatively small thickness (5–10 nm). This phenomenon is later explained again in Section 3.3.

The same plate appearance, though much less aggregated, is also displayed in the NH_3_·H_2_O samples, which can be seen in Figure 3. Furthermore, it can be seen from Figures 2A and 3A,C that the nanoplates synthesized by the two pathways both exhibit face-to-face self-assembly to form columns of Cu_2_S plates (marked by white rectangles) under certain temperatures. This effect has previously been observed by Li et al. 2010,^26^ and it is understood that at small particle sizes, a reduction of the nanoparticle surface energy can occur by self-assembly of nanoplates into a columnar structure (which is best observed with nanoplates synthesized with NaOH at 20 °C from Figure 2A, also the smallest nanoplates in the data set).

Interestingly, one can observe from Figures 2 and 3 that there are more round than hexagonal-like nanoplates. This phenomenon has been observed in previous studies which report on making round Cu_2_S nanoplates.^27,28^ Specifically, Zhuang et al. 2008^27^ reported that in the case where there are only weak ligands within the system, the reaction rate will be higher and therefore allow the formation of circular nanocrystals. Given that our synthesis route does not use strong ligands such as polyvinylpyrrolidone, a similar outcome is plausible. In another paper by Liu et al. 2017,^28^ the authors discuss this further regarding systemic thermodynamics and kinetics. Particularly, they conclude that in their specific setting of making Cu_2_S nanocrystals from CuS, their specific combinations of ligands and solvents, minimizations of surface energy provide a driving force for the transition from hexagonal crystals to round circular crystals. We suspect that our synthesis route also demonstrates the necessary thermodynamic driving force to make the nanoplates into circular shape.

A twinning effect is also observed, the prevalence of which depends on the preparation conditions. Specifically, in Figure 3A, white ovals are used to indicate several plates that are twinned, in which a small perpendicular nub grows in the centerline of the main plate. This effect is also observed in a paper by Miller et al. 2021^29^ where Fe_2_GeS_4_ nanoparticles would assemble into triplets of twins (trillings). The stacked nanoparticles in that paper intersect in a 60° angle to form these trillings. In the case of Cu_2_S, we typically observed the twinned effect of two single plates intersecting at a 90° angle. Furthermore, we observe that at elevated temperatures (starting at 40°), nearly all of the nanoparticles are single-plate. To explain the twinned effect, Miller et al. 2021^29^ stated that twinning happens when the lattice parameters and/or angles of a crystal structure are close to values that would encompass a higher symmetry than described by the structure’s space group. Furthermore, twinned particles show up more if the solution is more concentrated as supersaturation is more likely to overcome the instability of the twinned boundaries. This reasoning then explains why twinned plates are less frequently observed than single plates because of the low concentration of the CuSO_4_ precursor (at only 5 mM). In their paper, Miller et al. 2021 also mentioned that the use of alkylamine solvents could provide amide species that act as a Bronsted base, allowing highly reactive metal alkylamide intermediates. Subsequently, these reactive intermediates could provide the opportunity for the formation of twinned nanoparticles. In our paper, although an alkylamine solvent is not used, the fact that the twinned effect does not show up in any NaOH runs suggests that the copper–ammonia complex may also play a similar role to the amide species.

In our samples, we observed two unique trends with regard to size variance depending on the base used. For samples that are synthesized by NaOH, the plate size generally increases with respect to an increase in temperature. On the other hand, the plates size for Cu_2_S plates synthesized by NH_3_·H_2_O shows a more complicated trend, which can be better visualized in Figure 5. It can be first observed for the NH_3_·H_2_O samples (blue curve is shown in Figure 5) that the plate size increases from around 80 to 120 nm as the solution temperature is increased from 10 to 20 °C, after which the plate size decreases by roughly 30 nm until 40 °C where the diameter of the plates stays relatively constant (at 60 nm) until 60 °C. Nevertheless, it can be observed from the SEM micrograph of Figure 3C that the thickness of the plates has dropped to around 10 nm rather than a typical 20 nm range when heating is increased from 40 to 60 °C.

The nanoplates’ average diameter modulation can also be observed from the integration of the nanoplates in the ANF scaffold. Experimentally, we observed that all of the nanoplates synthesized at or above 40° with NaOH can be easily removed from the surface of the ANF after drying. Given that the pore size of the ANF is around 200–500 nm (Figure S1, Supporting Information section, shows the SEM image for the ANF), this observation is supported by our observed nanoplate sizes as the nanoplates synthesized at the above temperatures are either larger than 100 nm (sometimes even larger than 400 nm) and/or very polydisperse. Thus, it is important to choose the suitable reaction temperature to produce nanoplates with a suitable size to incorporate correspondingly to a matrix system (especially porous matrix like ANF).

This difference in size modulation trends between the NaOH and NH_3_·H_2_O samples arises from the modification of surface chemistries in their respective systems. Specifically, when NH_3_·H_2_O is used, it has been reported that NH_3_ can complex with copper ions.^30,31^ Using the widely accepted model of LaMer’s burst nucleation mechanism^32^ to understand the initial nucleation and growth of these nanoplates, the complexation between the NH_3_ and Cu ions can influence the sizes of the final nanoplates as follows. The copper ions are more stable because complexation before reduction by N_2_H_4_ leads to a slower nucleation rate in comparison to NaOH where no additional complexation is introduced. Thus, near room temperature (20 °C as shown in Figure 5), the starting size of the NH_3_·H_2_O colloidal particles is at ∼120 nm compared to ∼30 nm for nanoparticles derived from the NaOH solution because fewer nuclei eventually lead to larger particles following the growth stage. For low temperatures (∼10 °C) near the freezing point of the water solvent, complexation between NH_3_·H_2_O and Cu is minimized, and smaller particles are expected.

**Figure 4:**
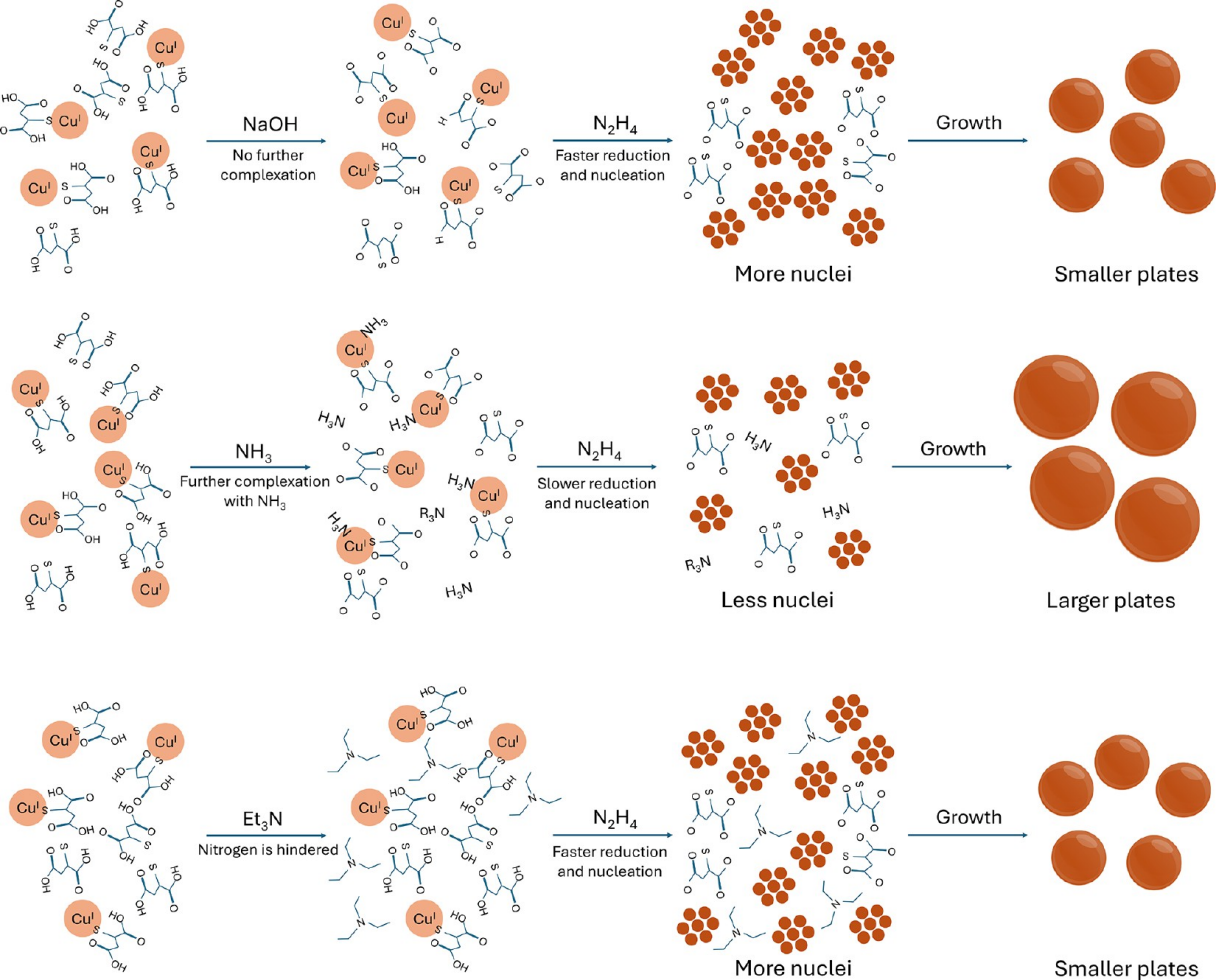
Illustrations of how the pH modifier can affect the nanoplates’ size in the reported synthesis route when (A) NaOH is used, (B) when NH_3_·H_2_O is used, and (C) when Et_3_N is used.

For temperature excursions above room temperature to 40 °C, when heating is introduced, NH_3_ can decomplex more rapidly from the copper ions. In fact, the boiling point of 25% ammonia solution is 37.7 °C. In either mechanism, the nucleation rate will increase as now the ions are less hindered for assembly and consequently the particle size will decrease as heating is increased. For NaOH, since there is no additional complexation introduced, heat will merely increase the kinetic energy of the system which will favor the growth of nanoplates. Consequently, the plates size increases in consonance with the increase in reaction temperature. This hypothesis is especially supported by experimental observations of color change that are described in the Experimental Details section. The NaOH solution darkens more rapidly due to faster nanoparticle growth. To ease visualization, Figure 4 is included as a diagram that shows the illustration for the mechanism.

**Figure 5:**
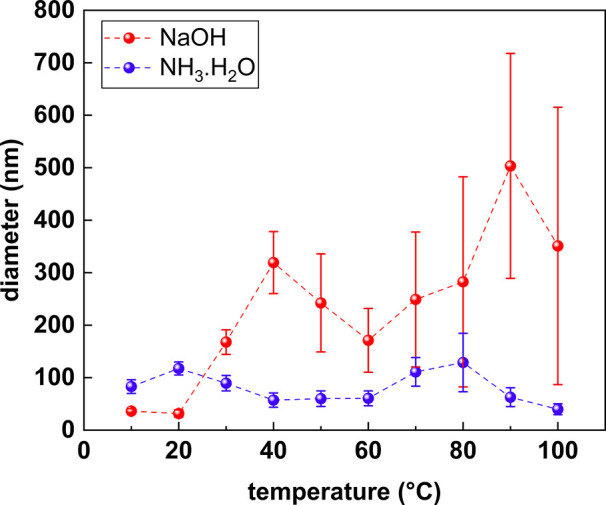
Overall trend for size modulation of Cu_2_S nanoplates by reaction temperature variation. The standard deviations for NaOH samples are 4 nm at 10 °C and 7 nm at 20 °C.

This complexation mechanism extends to explain the further particle size changes occurring in the NH_3_·H_2_O samples from 40 to 80 °C. The decomplexation effect can reach a saturated rate, in this case at 40 °C, as there is no further decrease in particle diameter. Since the decomplexation rate is now saturated, the elevated heating will soon favor the growth stage again just like the case of NaOH samples, and this can induce particle size growth that is observed between 60 and 80 °C. Interestingly, Figure 5 shows that the particle diameter diminishes starting at 90 °C. Given that such an observation is observed at a near boiling point temperature in a system where alkanethiol reagent is used, the trend can then be explained by the digestive ripening mechanism, which is the contrast of the Oswalt ripening mechanism. In the digestive ripening mechanism, the larger particles are broken down (redissolution) by a digestive ripening agent to create monomers for the smaller particles to grow (redeposition). In this mechanism, Sidhaye and Prasad in their 2011 review paper^33^ of the mechanism mentioned that alkanethiols are typically used as the digestive ripening agents thanks to their high affinity toward the metal surface. The authors also touched on how most of the digestive ripening reactions run at the boiling point of the solvent so that the larger particles can be etched out by the digestive ripening agent. Later in 2017, Shimpi et al.^34^ later explained the reason for heating as (a) because the melting point of nanoparticles are generally lower than that of the bulk, heating will increase the fluidity of the nanoparticles that would allow rearrangement of atoms, and (b) heating can alter the movement of different species within the system—the diffusion rate, the solubility, etc.—which eventually establishes the conditions to trigger digestive ripening. Examining the data, we can observe that the size modulation trend in Figure 5 aligns with this mechanism. First, at 80 °C, the system shows a record high polydispersity with the particles diameter equal to 130 ± 60 nm, indicating the start of digestive ripening. Subsequently, at 90 and 100 °C, both particles average diameter and diameter standard deviation decrease, setting a record low standard deviation at 100 °C with 40 ± 10 nm.

Even a few data points of the NaOH runs can also be explained by this mechanism. At 50 °C, a sudden drop in particle size can be seen in the red curve from Figure 5 for the NaOH sample with the value of 240 ± 90 nm. This is followed, at 60 °C, with another decrease in average diameter but this time with a much smaller standard deviation. It is worth noting that digesting ripening is in fact observed at lower than boiling points temperature, as Lin et al. 2010^35^ has observed digestive ripening for gold nanoparticles at room temperature. After the 60 °C mark, the size-temperature trend reverses for the NaOH samples. These results are again observed in the literature, where for instance, Sahu and Prasad in 2013^36^ reported an increase in both size and polydispersity after the initial narrowing in the size distribution of gold nanoparticles in that case, in which digestive ripening with hexadecanthiol with increased temperature is observed, which even lead to precipitation upon prolonged heating. The precipitation observations are also observed in our case, where all the NaOH samples that run above 50 °C would yield black colloids with precipitation at the bottom of the flask after the reaction is completed, a behavior mirrored in observations in Shimpi et al. 2017.^34^ After the digestive ripening effect has been achieved, further heating of the system will lead to interparticle coalescence due to both the loss of ligand coverage on the surfaces of the particles and the Ostwald ripening effect. This possibly explains why most of the nanoplates aggregate at high (near boiling point) reaction temperatures for syntheses with either bases aside from the typical explanation of increased kinetic energy, diffusion, or thermal fluctuations.

The overall size modulation trend of each case is presented in Figure 5, whereas additional SEM images for the temperature variation can be found in the Supporting Information section from Figures S3–S22.

To verify the proposed mechanism of NH_3_ complexation that governs the morphological changes of the nanoplates, another Cu_2_S nanoplate synthesis is conducted at 25 °C for 60 min using triethylamine (Et_3_N). This amine’s nitrogen, unlike NH_3_·H_2_O, is hindered by three ethyl groups which makes it theoretically impossible to complex with the copper ions. Thus, one could expect the nanoplates synthesized using this base will be small, comparable to those of the synthesis route using NaOH where no complexation occurred.

**Figure 6:**
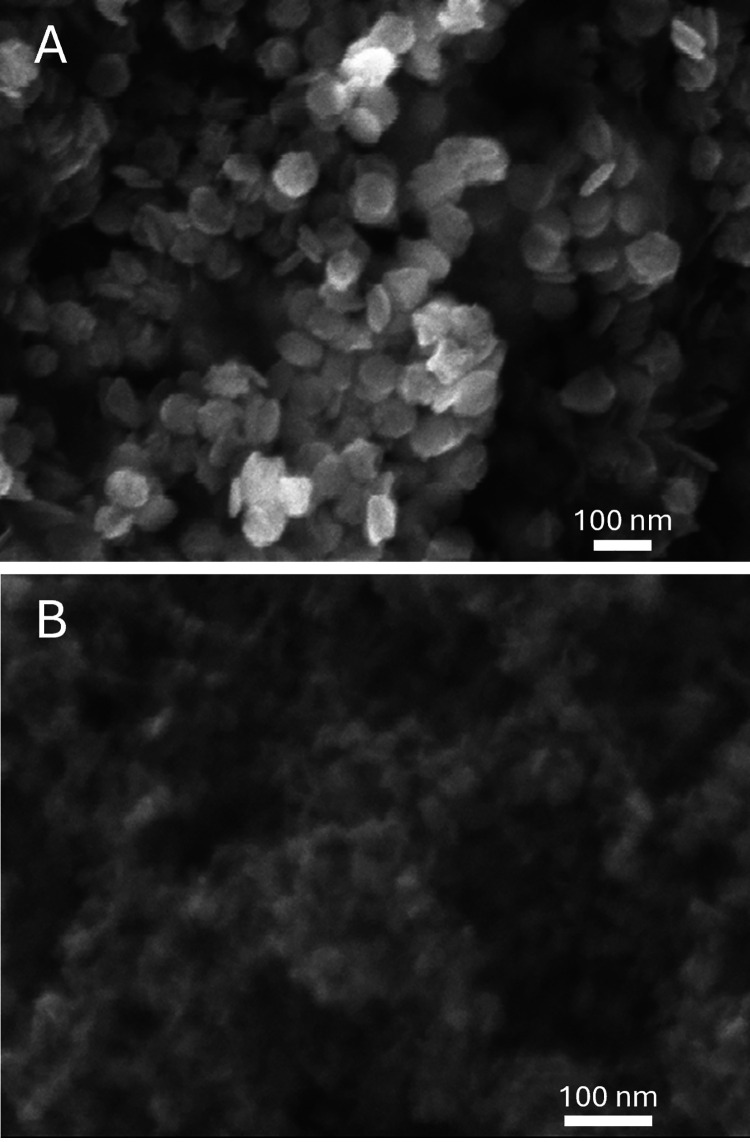
Cu_2_S nanoplates synthesized at 25 °C using 0.064 mL of N_2_H_4_ with (A) NH_3_·H_2_O as the base and (B) Et_3_N as the base.

As can be seen from Figure 6, the Cu_2_S nanoplates’ diameters are well-aligned with the proposed mechanism and hypothesis. Particularly, the average diameter of the sample synthesized with Et_3_N is significantly smaller (∼10 nm) than that of the sample synthesized with NH_3_·H_2_O (∼80 nm). This result confirms an interesting finding that the structure of the pH modifier itself can be manipulated to tune the size of the Cu_2_S nanoplates in chemical reduction synthesis. To support these results, in the Supporting Information section, Figure S29 shows additional SEM image for Cu_2_S sample synthesized with Et_3_N; Table S3 shows the EDS ZAF quantification table for Cu_2_S sample synthesized with Et_3_N, while Figure S33 shows the EDS elemental mapping for Cu_2_S sample synthesized with Et_3_N; and Figures S39 and S42, show additional raw and fitted XRD graphs for Cu_2_S sample synthesized with Et_3_N, respectively.

### 3.2 Effect of Changing the Reducing Agent (N_2_H_4_) Concentration

**Figure 7:**
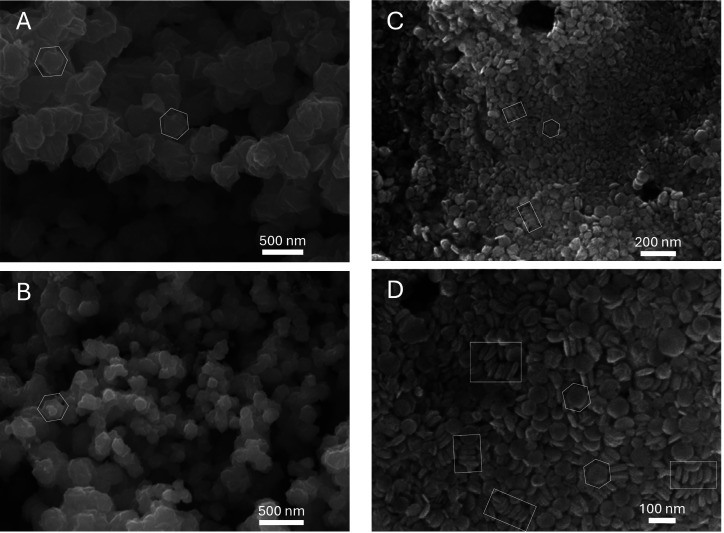
SEM images of Cu_2_S samples synthesized at 40 °C using (A) 0.064 mL of N_2_H_4_ with NaOH as the base; (B) 0.64 mL of N_2_H_4_ with NaOH as the base; (C) 0.064 mL of N_2_H_4_ with NH_3_·H_2_O as the base; (D) 0.64 mL of N_2_H_4_ with NH_3_·H_2_O as the base; note that Figure (A) in this section is the same as Figure 2. (B,C) in this section is the same as Figure 3. (B). White hexagons indicate the shape of the plates, whereas white rectangles indicate the self-assembly of the plates.

**Figure 8:**
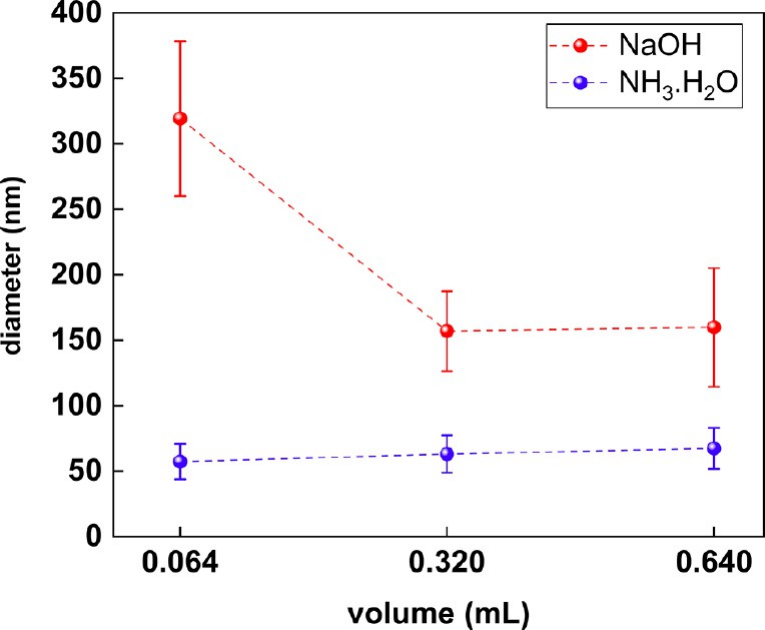
Overall trend for size modulation of Cu_2_S nanoplates by reducing agent concentration variation.

For those recipe changes that focused on the impact of reducing agent concentration, the reaction time for the colloidal solutions was again 60 min in all cases. In order to gauge the effect of changes to the concentration of the hydrazine reducing agent, the reaction temperature is held constant at 40 °C for all synthesis (NaOH and NH_3_·H_2_O). The amount of N_2_H_4_ used was then varied from 0.064 to 0.32 and 0.64 mL, which is equivalent to 1, 5, and 10 times the molar amount of the 0.08 g CuSO_4_ precursor. Likewise, the images and plotting are reported in Figures 7 and 8.

Unlike size modulation using heat, the variation of reducing agent concentration for the Cu_2_S nanoplate synthesis yields little to no change in the plate size. Similar to variations by heat, the trends of size modulation are different between NaOH and NH_3_·H_2_O when the N_2_H_4_ concentration is increased from 1, 5, and 10 times on a molar basis. For the NaOH samples, the average diameter of the plates drops substantially from an average of 319 nm to an average of 157 nm when the N_2_H_4_ concentration is increased from 1× to 5×. At 10× N_2_H_4_ concentration, the plates size remains similar (around 160 nm) to the plates size at 5× N_2_H_4_ concentration, suggesting that the reducing agent is near saturation at 5× N_2_H_4_ concentration. The samples synthesized with NH_3_·H_2_O as the base have a small increase of about 5 nm from 1 to 5 and 10× the N_2_H_4_ concentration. However, given that the standard deviation for the average size calculation is above 13 nm for all cases, it is inconclusive that there is actually an increase in size with an increase in N_2_H_4_ concentration.

Generally, literature suggests that increasing the amount of reducing agent decreases the particle size.^30,37^ The rationale is that when a reducing agent is introduced quickly at the beginning of the reaction, more nuclei will be formed if there is more reducing agent. As a result, a vast number of nuclei will yield smaller particles at the end of the growth stage, whereas a smaller number of nuclei will yield larger particles at the end of the growth stage. With NaOH, the results observed align with the above rationale. For the samples that are run with NH_3_·H_2_O, the results of no size modulation can be explained that the rate-determining step of this nanoplates synthesis pathway is unrelated to the amount of reducing agent used given that there is enough amount used to fully reduce the copper. Therefore, the rate-determining step could be dependent on the availability of the precursors (which are kept fixed in all of the variations) or on the diffusion capability of the system (which is related to heating). As explicated in the previous section, this system is heavily dependent on the reaction temperature. When surface chemistry related to NH_3_·H_2_O is reconsidered, this hypothesis is further supported. Given that the complexation–decomplexation of NH_3_ to copper ions are crucial during the nucleation stage of the system, this step could be, in fact, the rate-determining step. Therefore, varying the reducing agent concentration might not substantially alter the solution chemistry.

The overall size modulation trend of each case is presented in Figure 8, whereas the additional SEM images for the reducing agent concentration can be found in the Supporting Information section from Figures S23–S26.

### 3.3 Direct Comparison of the Cu_2_S Nanoplate Synthesized by NaOH and NH_3_·H_2_O Using TEM

To better compare the structural differences of Cu_2_S nanoplate synthesized by the two bases, TEM images are taken for experiments with NaOH and NH_3_·H_2_O at the temperature mark where the average plate diameter are closest to each other given that 0.064 mL of N_2_H_4_ is used. The data in Section 3.1 show that this temperature mark is 25 °C.

**Figure 9:**
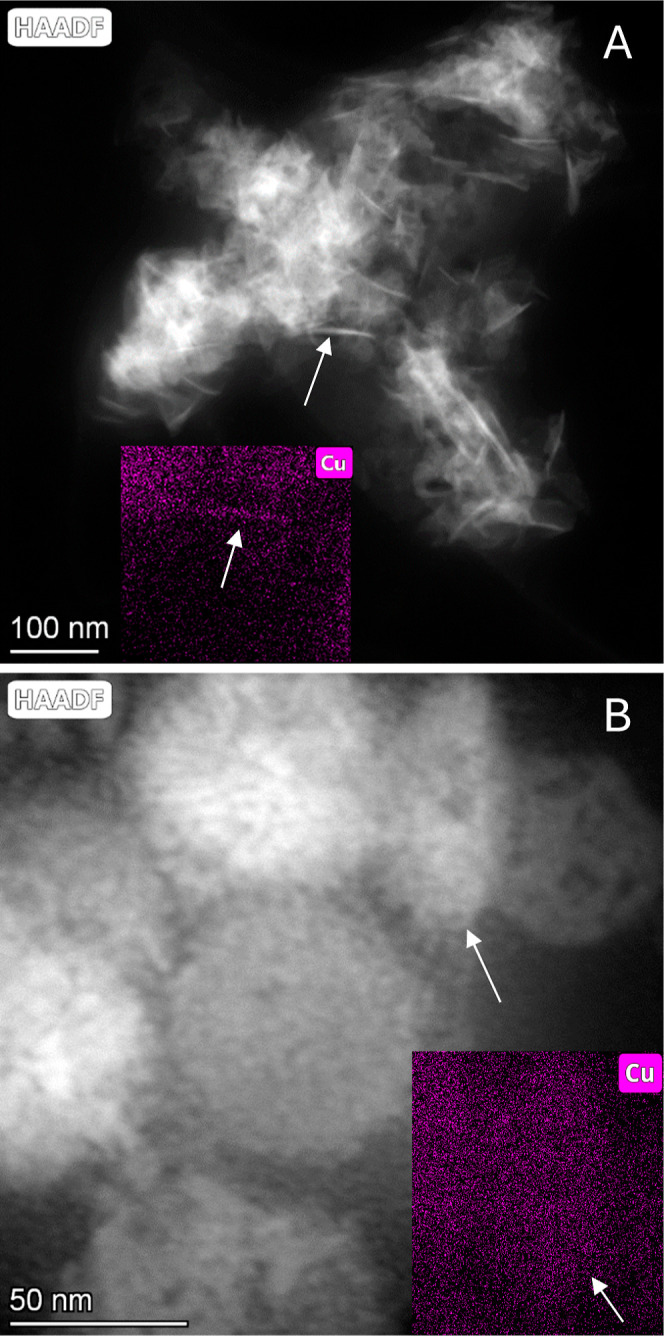
Cu_2_S nanoplates synthesized at 25 °C using 0.064 mL of N_2_H_4_ with (A) NaOH as the base and (B) NH_3_·H_2_O as the base. The arrows are to indicate the two plates that are being compared. The inset of both images contains an EDS elemental mappings of Cu.

From Figure 9A,B, nanoplates are oriented parallel to and perpendicular to the electron beam direction. Overall, the diameter of the plates is relatively homogeneous, at around 80 nm, for both NaOH and NH_3_·H_2_O syntheses. The biggest difference which can be immediately observed between two synthesis pathways is that the plates via NaOH are significantly thinner than the plates via NH_3_·H_2_O. The plate thickness of the NaOH sample is around 5 nm (Figure 9A), whereas the plate thickness of the NH_3_·H_2_O sample is around 20 nm (Figure 9B). This can then be used to explain the second major difference between the colloids which is the degree of aggregation between the two samples. Because the thickness of the NaOH sample is about 4 times smaller than that of the NH_3_·H_2_O sample given that the diameter is roughly the same, the surface-to-volume ratio of the nanoparticles within the NaOH colloidal solution are about 3 times larger than the surface-to-volume ratio derived from the NH_3_·H_2_O sample. Thus, the NaOH-synthesized plate possesses much higher surface energy than the NH_3_·H_2_O synthesized plate, and one therefore expects greater aggregation from the NaOH solution in order to minimize this energy, an expectation confirmed in both SEM and TEM micrographs.

As for why the NH_3_·H_2_O sample has a much larger thickness than the NaOH sample, we can attribute it to the complexation chemistry discussed in Section 3.1. Particularly, since NH_3_·H_2_O can complex with a Cu^2+^ ion to stabilize it, this stabilization could lead to a more controlled and layered growth, which leads to thicker nanoplates. The synthesis with NaOH, on the other hand, does not have this stabilization and as a result can occur more rapidly with less control. This hypothesis is likely, given that as mentioned in Section 3.1, we discussed that the copper–ammonia complex could be removed rapidly starting at 40 °C given that the boiling point of NH_3_·H_2_O 25% is 37.7 °C. When no NH_3_ is stabilizing the system, one observes that the nanoplates’ thickness of NH_3_·H_2_O samples reduces to around 10 nm at 60 °C.

The inset graphs of the TEM images in Figure 9 show that copper maps to these hexagonal-like features, either edge facing or surface facing. To support these results provided here, in the Supporting Information section, Figures S27 and S28 show additional SEM images of Cu_2_S samples synthesized with 0.064 mL of N_2_H_4_ (1×) at 25 °C using NaOH and NH_3_·H_2_O as the base, respectively; Figure S30 shows additional TEM images of Cu_2_S samples synthesized with 0.064 mL of N_2_H_4_ (1×) at 25 °C using NaOH and NH_3_·H_2_O as the base; Table S1 and Figure S31 show additional composition and phase analysis using EDS through ZAF quantification and elemental mapping, respectively, of Cu_2_S samples synthesized with 0.064 mL of N_2_H_4_ (1×) at 25 °C using NaOH; Table S2 and Figure S32 show additional composition and phase analysis using EDS through ZAF quantification and elemental mapping, respectively, of Cu_2_S samples synthesized with 0.064 mL of N_2_H_4_ (1×) at 25 °C using NH_3_·H_2_O; and Figures S39 and S42 show raw and fitted XRD graphs of the samples synthesized with 0.064 mL of N_2_H_4_ (1×) at 25 °C using NaOH, NH_3_·H_2_O, and Et_3_N, respectively. From Figure S30, it can also be seen that sulfur can still be spotted not only on the plates but also around the plates with lower intensity. There are at least three sources that can contribute to this S mapping, which are (a) a dithiol compound that is formed as a result of mercaptosuccinic acid auto-oxidation with copper catalyst, (b) mercaptosuccinic acid, which acts as ligands surrounding the Cu_2_S plates, and (c) excess MSA.

A percent yield calculation for these two samples is also conducted by measuring the actual retrieved product mass and divided by theoretical yield to give an estimation of the yield of all the samples reported in this paper. Specifically, the percent yield calculation for Cu_2_S nanoplates synthesized at 25 °C using 0.064 mL of N_2_H_4_ with NaOH is 77.00% and with NH_3_·H_2_O, the yield is 39.25%. Both numbers are high given that the yields reported by current syntheses are below 20%.^16^

### 3.4 Chemical Composition of the Cu_2_S Nanoplates

**Figure 10:**
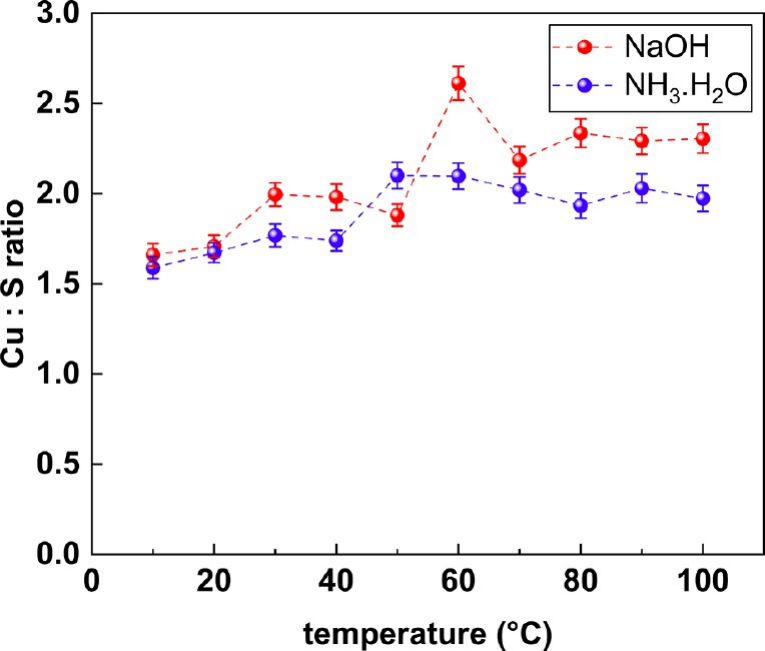
Cu to S ratio obtained from EDS of all the samples synthesized for the temperature variation test in Section 3.1.

The successful synthesis of Cu_2_S is first supported by EDS analysis of the material. The lines L_α_, L_β_, K_α_, and K_β_ of Cu and S all appear in the spectrum. Based on ZAF quantification, the Cu to S ratio is very close to a 1 to 2 ratio, with a small atom percentage of O (typically less than 10% atomic) which could be attributed to the oxygen atom in the carbonyl group of polyamides which are the building blocks of ANF. Figure 10 shows a plot of the Cu to S ratio of all Cu_2_S samples that have been produced during the temperature variation test. In the Supporting Information section, Figures S31–S33 show three additional representative EDS elemental mapping spectra of the Cu_2_S samples synthesized at 25 °C, while Tables S1–S3 show three additional representative ZAF quantification tables from the samples synthesized at 25 °C.

One can observe from Figure 10 that all of the Cu to S ratios typically lie between the 1.5 to 2.5 range, with a higher Cu to S ratio in samples that are synthesized at a higher temperature. This is expected given that all the samples react for the same amount of time (60 min), so the systems which have been provided with more energy will be able to reduce more copper. Although all the Cu to S ratios shown in Figure 10 are around 2:1, further investigation of the material crystallinity using XRD is still necessary due to the multiple metastable phases involving copper(I) sulfide occurred including Cu_7_S_4_ (anilite),^38,39^ Cu_9_S_5_ (digenite),^40^ Cu_58_S_32_ (roxbyite),^41^ and Cu_31_S_16_ (djurleite)^39^ that are all very close to a 2 to 1 ratio.

### 3.5 Crystalline Structure of the Cu_2_S Nanoplates

**Figure 11:**
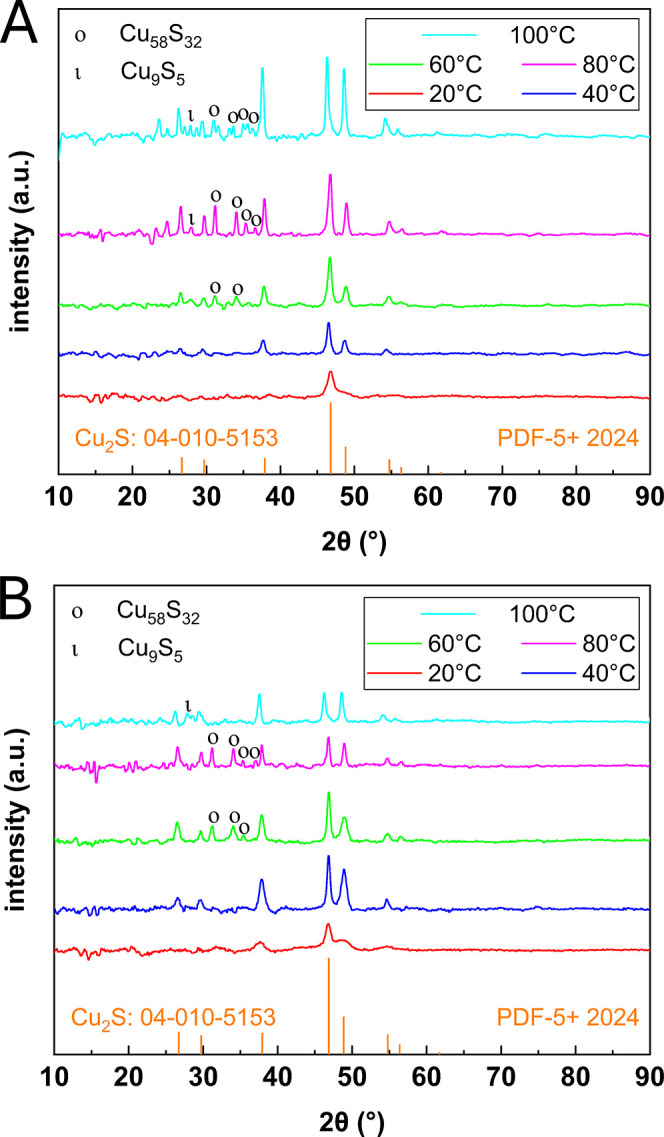
XRD analysis for Cu_2_S synthesized using 0.064 mL of N_2_H_4_ (1×) with (A) NaOH as the base and (B) NH_3_·H_2_O as the base at 20, 40, 60, 80, and 100 °C.

Further investigation of the Cu_2_S material is carried out by XRD. As can be seen from Figure 11, all measured XRD spectra possess multiple diffraction signals that can be attributed to the typical high chalcocite structure without showing any other signals that could be indicative of cupric oxide. Nevertheless, at elevated temperatures from 60 to 100 °C, trace amounts of Cu_9_S_5_ digenite (01–078–4724: PDF-5+ 2024) with a peak at about 28.02° (1 1 1) and Cu_58_S_32_ roxbyite (00–064–0278: PDF-5+ 2024) with peaks at around 31.15° (1–3 −4), 33.91° (5 1 0), 35.35° (5 0 2), and 36.42° (2 5–1) can be observed. The rest of the peaks, though, are very indicative of high Cu_2_S high chalcocite. Specifically, the several peaks at around 37.77° (1 0 2), 46.67° (1 1 0), 48.83° (1 0 3), and 55.25° (1 1 2), 56.66° (2 0 1), and 61.83° (2 0 2) are very unique to chalcocite (04–010–5153: PDF-5+ 2024). Furthermore, when the effect of reaction temperature is further inspected, it could be seen that the peak intensity generally increases with temperature as the crystallinity of the nanocrystallites is improved. This trend is true for all data except the 80 and 100 °C distributions of the NH_3_·H_2_O experiments where the peaks show a decrease in intensity. Again, this can be explained by the fact that the particles are actually getting smaller starting at 80 °C due to the digestive ripening effect.

Overall, this synthesis pathway produces relatively pure Cu_2_S high chalcocite, particularly at temperatures below 50 °C. Likewise, only two fitted XRD analysis spectra of the temperature variation runs are shown here. In the Supporting Information, Figures S35–S38 show additional raw XRD analysis for the synthesized Cu_2_S samples while Figures S40 and S41 show additional fitted XRD analysis for the synthesized Cu_2_S samples.

### 3.6 Optical Properties

**Figure 12:**
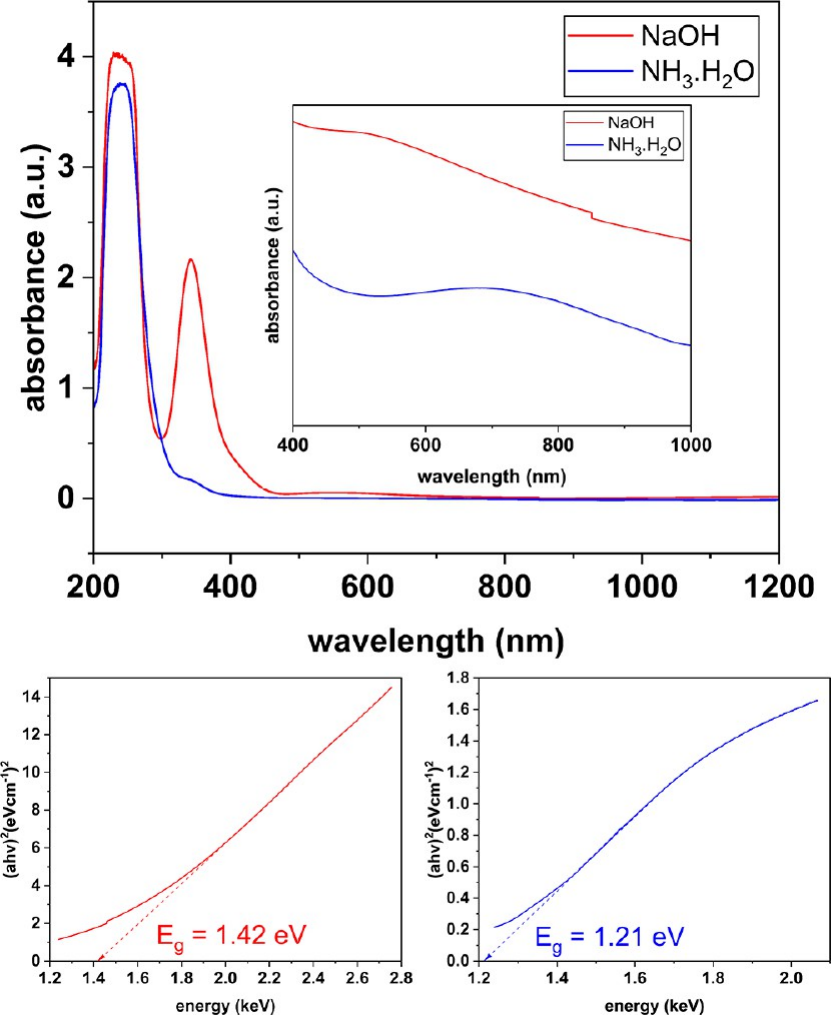
UV–vis–NIR spectrum of Cu_2_S nanoplates synthesized at 25 °C using 0.064 mL of N_2_H_4_ (1×) with NaOH and NH_3_·H_2_O as the base, along with the associated Tauc plots.

Aside from the characterization of the Cu_2_S nanoplates, the overall optical properties of the synthesized material have been investigated using a UV–vis–NIR spectrophotometer. As shown in Figure 12, the UV–vis–NIR spectra of two representative samples, which are the samples synthesized using 0.064 mL of N_2_H_4_ at 25 °C with both bases, show high absorption at around 240 and 340 nm. Experimentally, the high absorption at around 240 nm first appears when MSA is added to the CuSO_4_ solution, whereas the high absorption at around 340 nm appears when the bases are added. The high absorption at around 340 nm is likely due to the Cu–OH interaction. In the case of NH_3_·H_2_O, because NH_3_ is also complexing with Cu in competition with the Cu–OH interaction, Figure 12 shows that the absorption at 340 nm is far less intense than that in the case of NaOH. Experimentally, if the colloids are left overnight, the absorption at 340 nm will disappear completely, possibly indicating the completion of Cu_2_S reduction.

Additionally, when a concentrated sample is used during the UV–vis–NIR measurement with a focus on the wavelength interval between 400 and 1000 nm, clear, broad exciton peaks can be spotted in the UV–vis–NIR graphs of the two samples. One can also observe that this exciton NaOH-derived sample has a peak that is blue-shifted relative to the colloid derived from the NH_3_·H_2_O base reaction. This is consistent with the differences in the depths of the nanoplates, as shown in Figure 9. Though the diameter of the nanoplates from the two runs are similar, the thickness of the sample synthesized by NaOH is 5 nm, compared to 20 nm of the sample synthesized by NH_3_·H_2_O. This is crucial as the Bohr radius of Cu_2_S is 5 nm^42^ and thus the sample synthesized with NaOH can demonstrate a stronger quantum confinement effect leading to an increase in the band gap energy of the material.

As mentioned in the Introduction section, in the bulk, the band gap energy of Cu_2_S is about 1.2 eV. To examine the band gap energy of the synthesized samples, the UV–vis–NIR graph is converted to the Tauc Plot^43^ given that Cu_2_S is a direct band gap material.^42^ This method assumes that the energy-dependent absorption α can be represented by the following equation
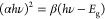
where *h* is the Planck constant, ν is the photon’s frequency, *E*_g_ is the band gap energy, and β is a constant.

The Tauc plot in Figure 12 shows that both samples exhibit a band gap energy that is well-aligned with the band gap energy of Cu_2_S. What is more, the fact that the band gap energy of the sample synthesized with NaOH (1.42 eV) is larger than that of the sample synthesized with NH_3_·H_2_O (1.21 eV) confirms the observation that the nanoplates with a thickness of 5 nm do show a quantum confinement effect. With these band gaps derived from the Tauc plot, in the Supporting Information, Figure S43 shows an energy band diagram for our synthesized Cu_2_S samples.

## 4 Conclusions

Via a three-step, one-pot aqueous chemical reduction synthesis, Cu_2_S nanoplates with tunable sizes between 30 and 300 nm are synthesized successfully using two different bases: NaOH and NH_3_·H_2_O. The structures of the nanoplates prepared by each base produce both structural differences and size modulation differences. Overall, this article reports an efficient synthesis pathway to produce Cu_2_S nanoplates at ambient temperatures condition as a potential replacement for typical thermolysis synthesis of Cu_2_S. Regarding a comparison between the use of NaOH and NH_3_·H_2_O as the base for this synthesis, it can be seen that the use of NH_3_·H_2_O is preferred as it produces more-defined nanoplate structures. Nevertheless, from an environmental consideration standpoint, it is worth noting that NH_3_·H_2_O is more environmentally hazardous compared to NaOH. In conclusion, this paper offers extensive studies for the synthesis and size modulation of technologically relevant Cu_2_S.

## 5 Outlook and Implications

**Figure 13:**
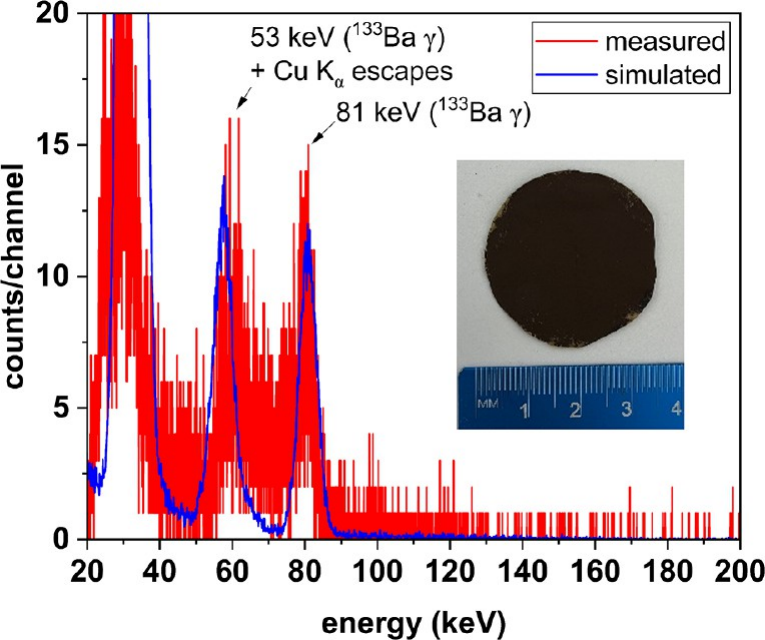
Gamma-ray spectrum from Cu_2_S/ANF synthesized at 25 °C using 0.064 mL of N_2_H_4_ (1×) with NaOH as the base. The inset picture shows the sample tested.

ANF can be used as a scaffold upon which Cu_2_S nanoplates can be chemically bonded and thereby incorporated into percolating charge-transport networks (Figure S44, Supporting Information section, shows the macroscale images of the composites from Cu_2_S synthesized with 0.064 mL of N_2_H_4_ at 25 °C using NaOH and NH_3_·H_2_O), a functionality that is important for the production of lightweight, free-standing sensors, battery electrodes, and radiation shields. In battery configurations, one can surface load the ANF and abut the Cu_2_S side to the copper electrode in the battery cell while the unloaded ANF serves as the separator. When a heavily loaded thin-film solid is coupled to an electronic readout chain (Figure S45, Supporting Information section, shows additional image and diagram for the photonic response measurement setup), the photonic spectrum shown in Figure 13 can be observed. The distribution is collected when gamma-rays from radioactive isotope Barium-133 (^133^Ba) is impinged upon a Cu_2_S/ANF thin film synthesized using 0.064 mL of N_2_H_4_ (1x) at 25 °C with NaOH as the base. The thin film is lightweight with a thickness of only about 7 μm, and the density of the film is 1.470 g/cm^3^.

As shown in Figure 13, the film has detectable photonic response features with 53 and 81 keV, the two energies from ^133^Ba gamma-rays (and associated X-ray escape features). The energy resolution is 3.2 at 81 keV (4%). The measurement result well-matches a simulation conducted with the Monte Carlo N-Particle (MCNP6) nuclear transport code, in which the thickness of the simulated solid is increased to account for the improved stopping of charged particles that accompanies nanostructuring the material,^22^ as detailed in the Experimental Details section. A Cu_2_S/ANF circular film prepared via the NH_3_·H_2_O route (0.064 mL of N_2_H_4_ (1x) at 25 °C using NH_3_·H_2_O) had a low enough density (0.885 g/cm^3^) that no signals were observed, presumably because the network had a density below the charge-transport percolation limit.

Regarding the chemical synthesis of Cu_2_S, the authors believe that further investigations should be centered around the effect of different conditions on structure and size modulation such as the reaction time, pressure, and precursor ratio (MSA to CuSO_4_). This modulation study should specifically target syntheses at low temperatures as it has been shown in this paper that above 50 °C, the presence of impurity phases is enhanced. Additionally, the study of long chain ligands such as polyvinylpyrrolidone or cetyltrimethylammonium bromide may provide valuable insight to determine if aggregation can be prevented when using NaOH as the base for the synthesis or when extensive heating is introduced. For device integration, we also wish to better marry the linear Cu_2_S stacks that can result from self-assembly with the linear fibers within the scaffold. One could further study self-assembly methods to control the stacking of Cu_2_S nanoplates as well as how this stacking effect could change the properties of the material. The digestive ripening effect can also be further investigated as it shows a potential in producing monodisperse sub-50 nm nanoplates in this article.

## Supporting Information Available

Additional SEM and TEM images of ANF and Cu_2_S nanoplates formed at varying temperatures from 10 to 100 °C using varying concentrations of N_2_H_4_ from 0.064 to 0.64 mL with different pH modifiers (NaOH, NH_3_·H_2_O, and Et_3_N); size distribution measurements taken using a dynamic light scattering device Zetasizer Nano ZSP for Cu_2_S samples synthesized with 0.064 mL of N_2_H_4_ (1x) at 20 °C using NH_3_·H_2_O as the base; additional EDS elemental mapping graphs and ZAF quantification of Cu_2_S nanoplates formed at varying temperatures from 10 to 100 °C using varying concentrations of N_2_H_4_ from 0.064 to 0.64 mL with different pH modifiers (NaOH, NH_3_·H_2_O, and Et_3_N); additional raw and fitted XRD analysis graphs of Cu_2_S/ANF composites fabricated with Cu_2_S nanoplates formed at varying temperatures from 20 to 100 °C using varying concentrations of N_2_H_4_ from 0.064 to 0.64 mL with different pH modifiers (NaOH and NH_3_·H_2_O); energy band diagram for Cu_2_S nanoplates formed at 25 °C using 0.064 mL of N_2_H_4_ with different pH modifiers (NaOH and NH_3_·H_2_O); macroscale images of the Cu_2_S/ANF composites fabricated with Cu_2_S nanoplates formed at 25 °C using 0.064 mL of N_2_H_4_ with both NaOH and NH_3_·H_2_O; and experimental setup of the photonic response measurement for the Cu_2_S/ANF composite fabricated with Cu_2_S nanoplates formed at 25 °C using 0.064 mL of N_2_H_4_ with NaOH (PDF)

## Abbreviations

MSA: mercaptosuccinic acid
Et_3_N: triethylamine
DMSO: dimethyl sulfoxide
