# Supplementary material for: Size and Shape Modulation of Cu2S Nanoplates via Chemical Reduction with NaOH and NH3·H2O
Source: ACS Omega. 2024 Oct 30;9(47):46929–42. doi: 10.1021/acsomega.4c06316 (PMC11603283; doi:10.1021/acsomega.4c06316)
Supplement: Supplementary file 1 — ao4c06316_si_001.pdf [file ao4c06316_si_001.pdf]

*Supporting Information for:*

# Size and Shape Modulation of $Cu_2S$ Nanoplates via Chemical Reduction with NaOH and $NH_3.H_2O$

Vinh-Dien Le[1], Gabrielle J. Grey[1,2]*, Ill-hyuk Han[1,3], and Mark D. Hammig[1,2]

[1]College of Engineering, University of Michigan, Ann Arbor, MI 48109

[2]Amphionic LLC, Plymouth, MI 48170

[3]Department of Nuclear Engineering, Seoul National University, Seoul, South Korea, 08826

*Email: greyg@umich.edu

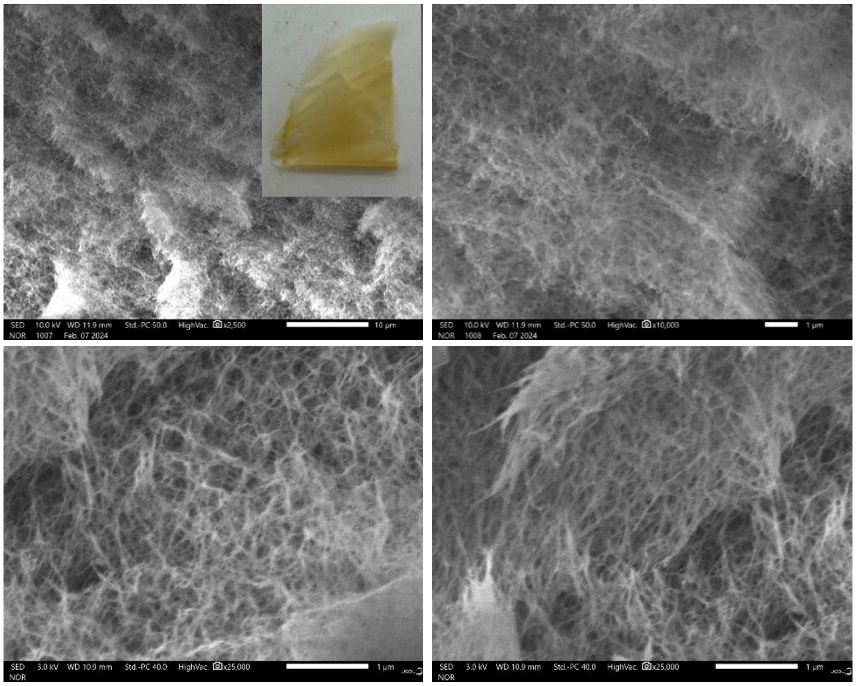

**Figure S1.** SEM images of aramid nanofiber 2% scaffold. An inset on one of the image is its the macroscale imaging

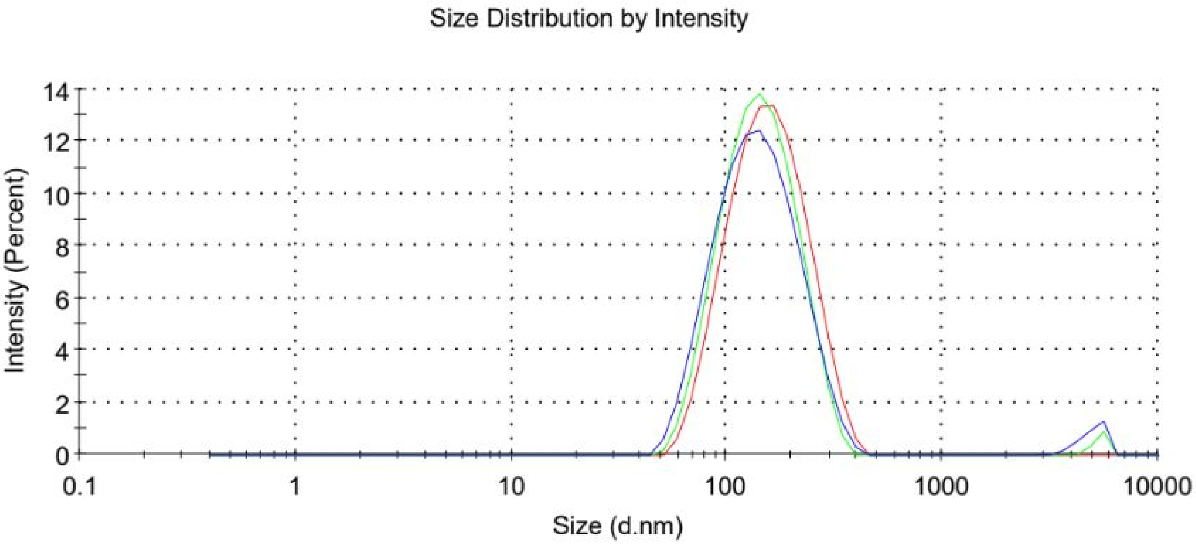


**Figure S2.** Size distribution measurements taken using a Dynamic Light Scattering device Zetasizer Nano ZSP for $Cu_2S$ samples synthesized with 0.064 mL $N_2H_4$ (1x) at 20°C using $NH_3.H_2O$ as the base shows the average diameter of 129.7 nm with a Polydisperse Index of 0.250. This result is relatively close to the manual measurements of nanoplates diameter which yield an average diameter of 118 nm ± 12 nm

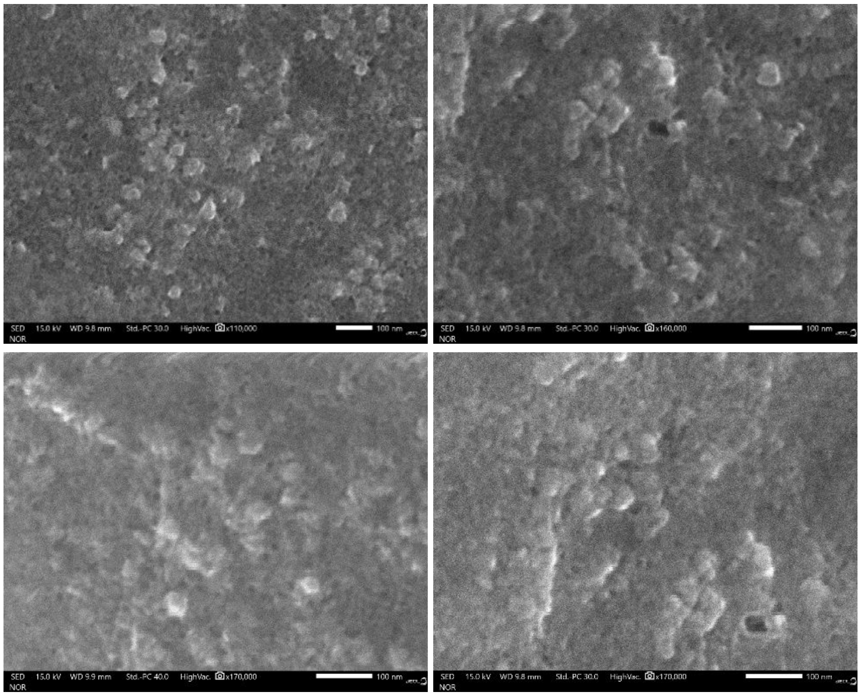

**Figure S3.** SEM images of $Cu_2S$ samples synthesized with 0.064 mL $N_2H_4$ (1x) at 10°C using NaOH as the base. Note that not many particles can be spotted from SEM imaging of this sample which indicates that not much particles are formed at such a low temperature

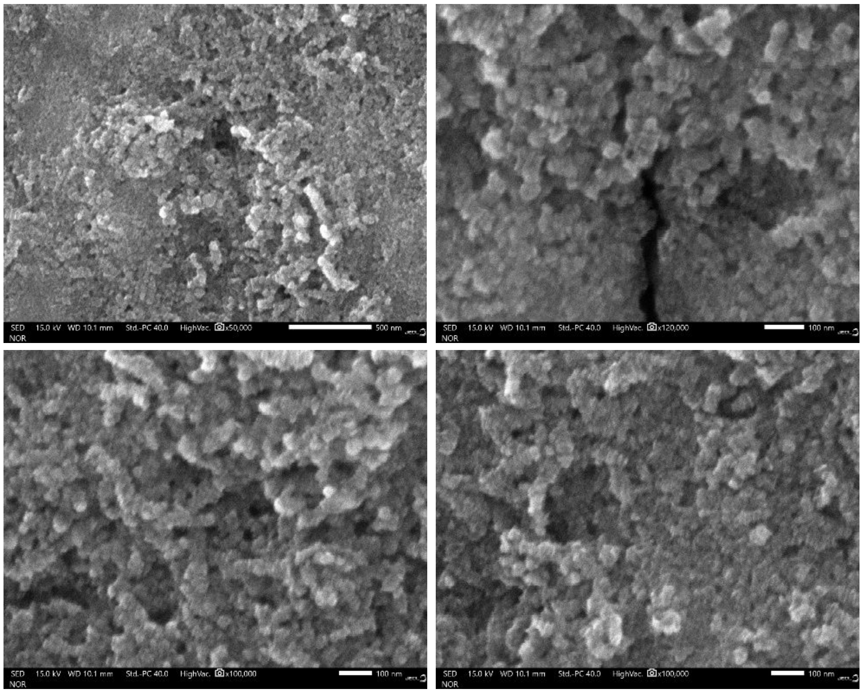

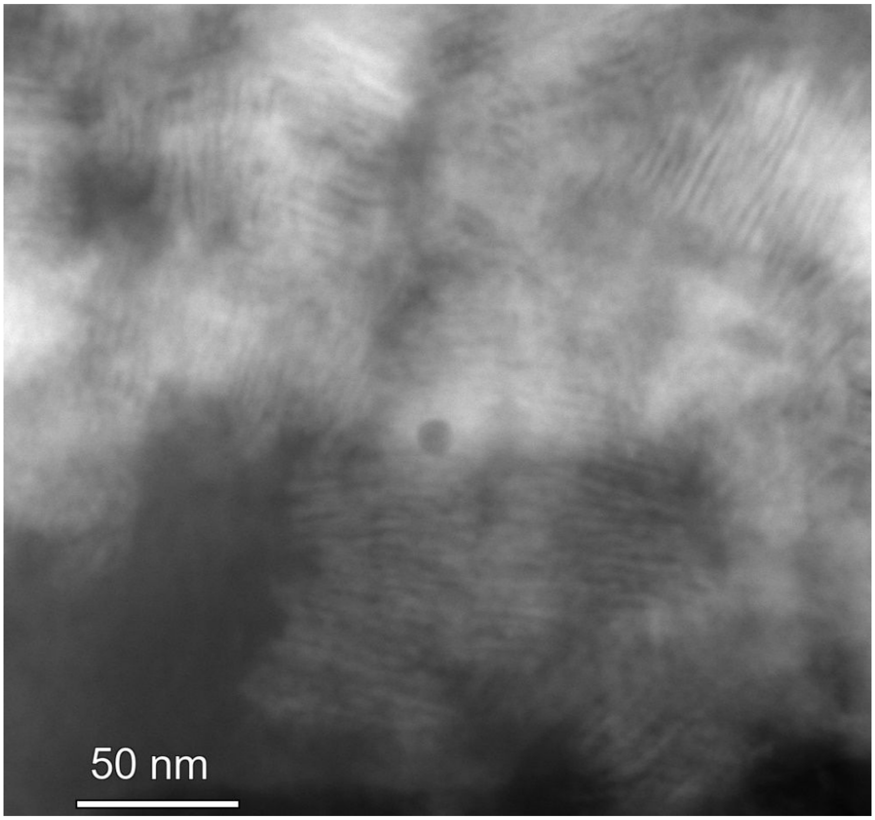


**Figure S4.** SEM and TEM images of $Cu_2S$ samples synthesized with 0.064 mL $N_2H_4$ (1x) at 20°C using NaOH as the base

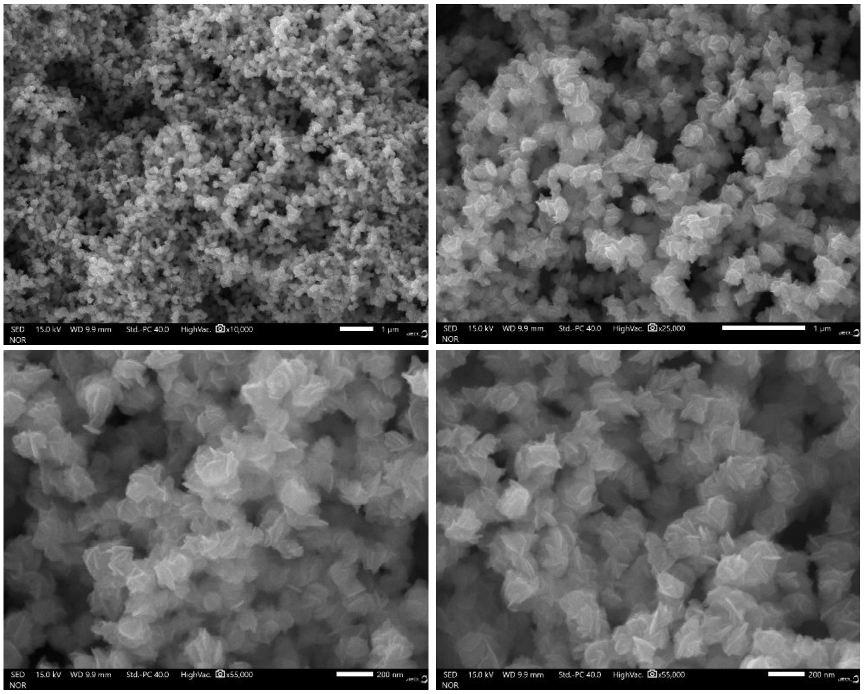

**Figure S5.** SEM images of $Cu_2S$ samples synthesized with 0.064 mL $N_2H_4$ (1x) at 30°C using NaOH as the base

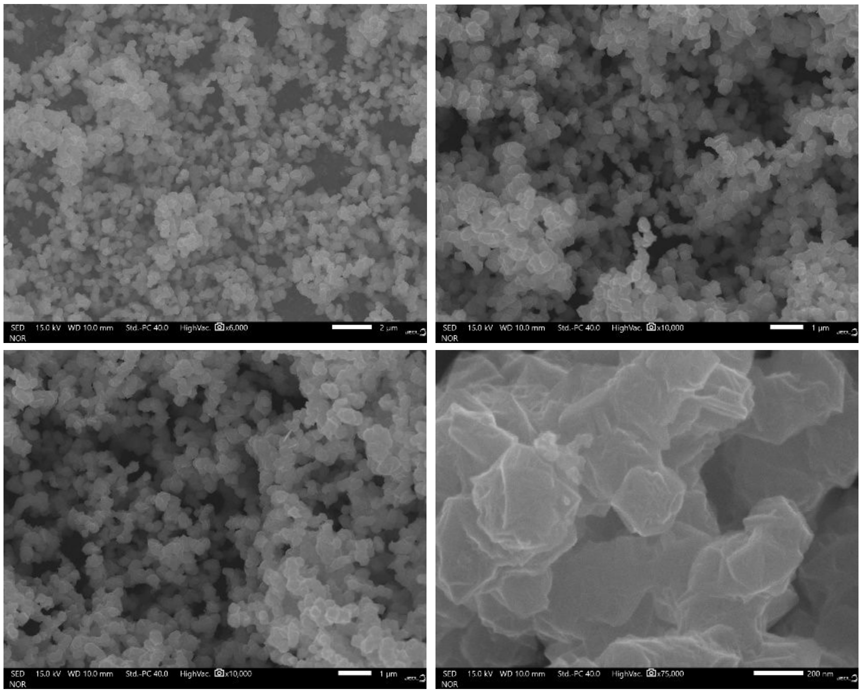

**Figure S6.** SEM images of $Cu_2S$ samples synthesized with 0.064 mL $N_2H_4$ (1x) at 40°C using NaOH as the base

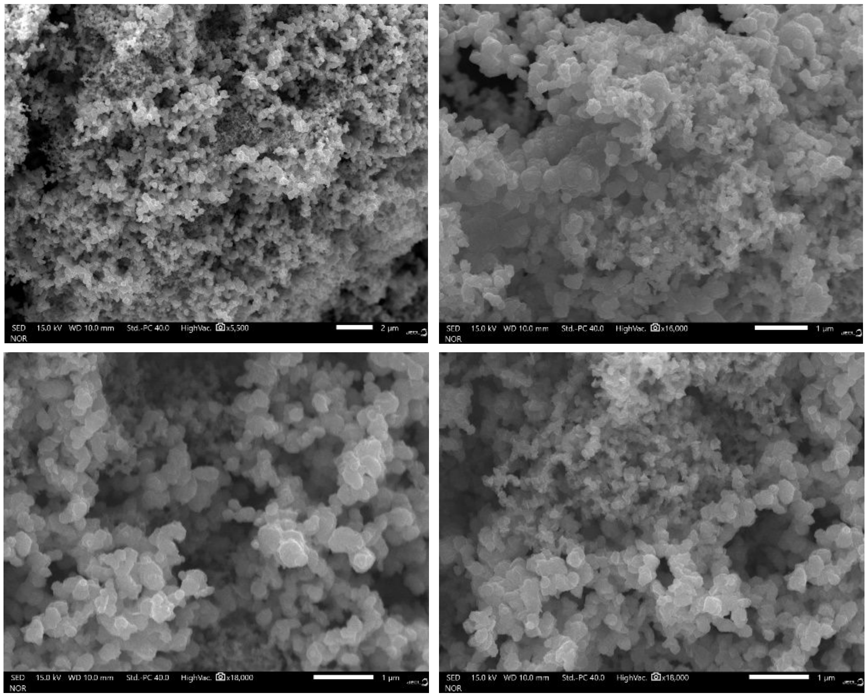

**Figure S7.** SEM images of $Cu_2S$ samples synthesized with 0.064 mL $N_2H_4$ (1x) at 50°C using NaOH as the base

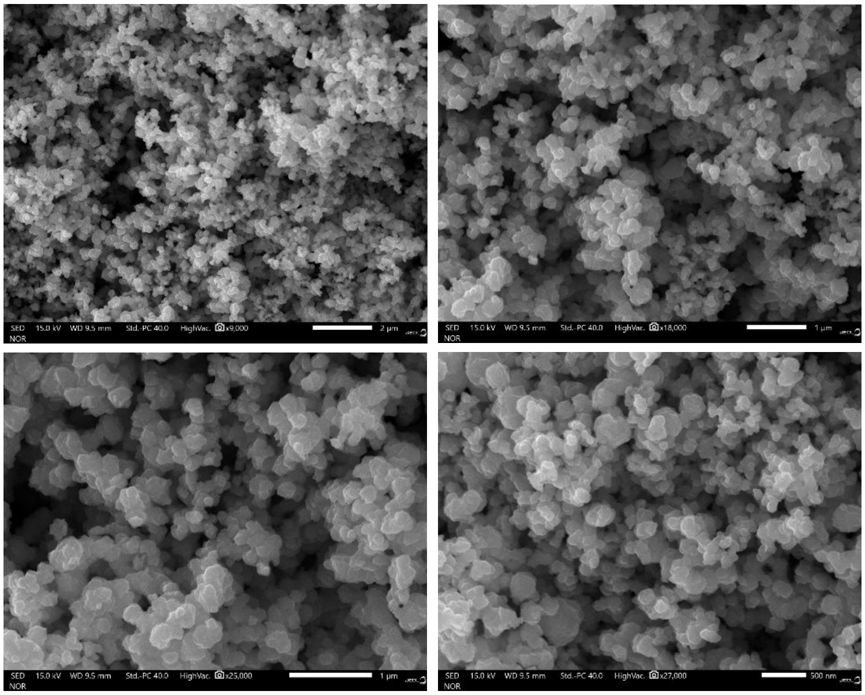


**Figure S8.** SEM images of $Cu_2S$ samples synthesized with 0.064 mL $N_2H_4$ (1x) at 60°C using NaOH as the base

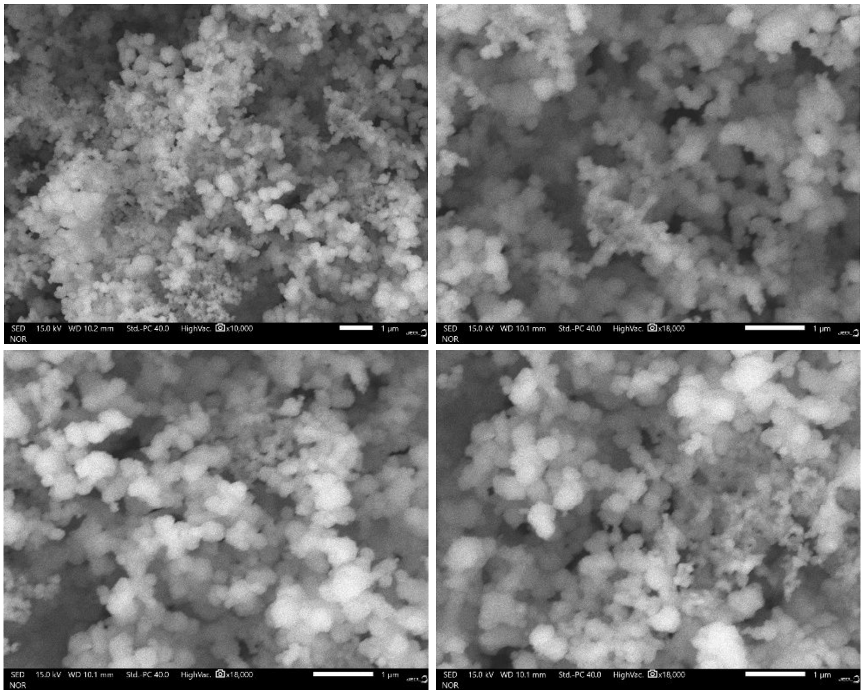


**Figure S9.** SEM images of $Cu_2S$ samples synthesized with 0.064 mL $N_2H_4$ (1x) at 70°C using NaOH as the base

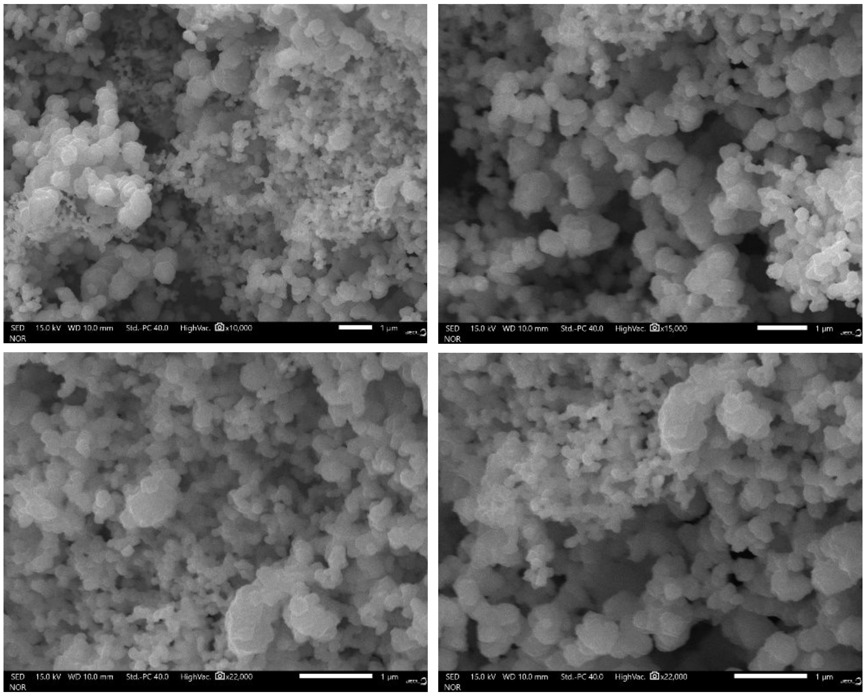

**Figure S10.** SEM images of $Cu_2S$ samples synthesized with 0.064 mL $N_2H_4$ (1x) at 80°C using NaOH as the base

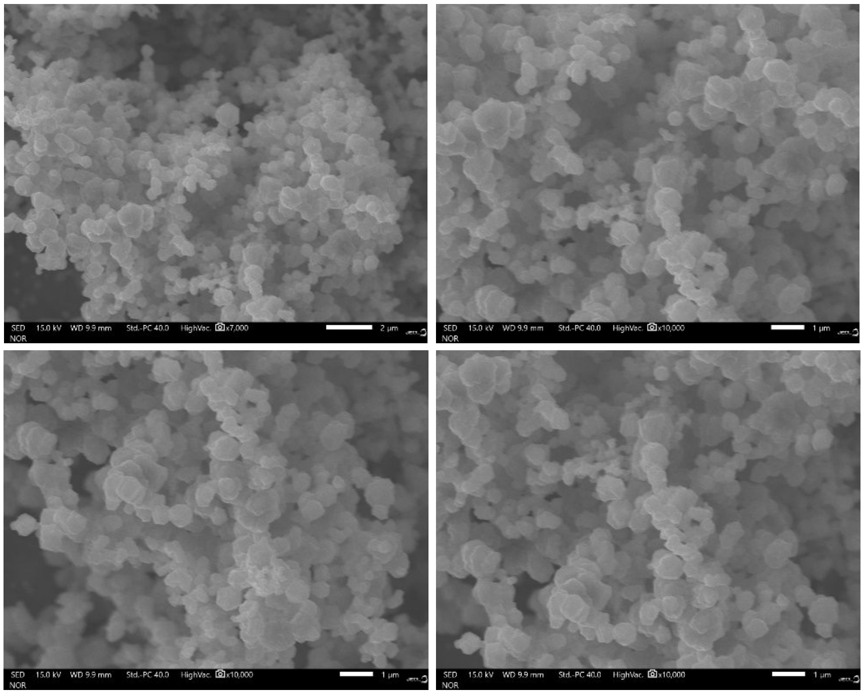

**Figure S11.** SEM images of $Cu_2S$ samples synthesized with 0.064 mL $N_2H_4$ (1x) at 90°C using NaOH as the base

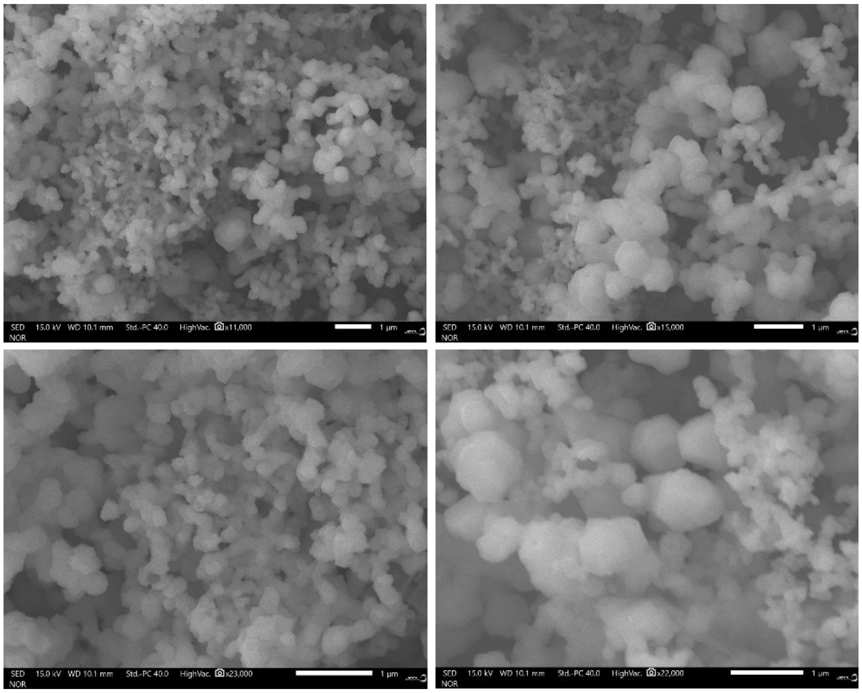

**Figure S12.** SEM images of $Cu_2S$ samples synthesized with 0.064 mL $N_2H_4$ (1x) at 100°C using NaOH as the base

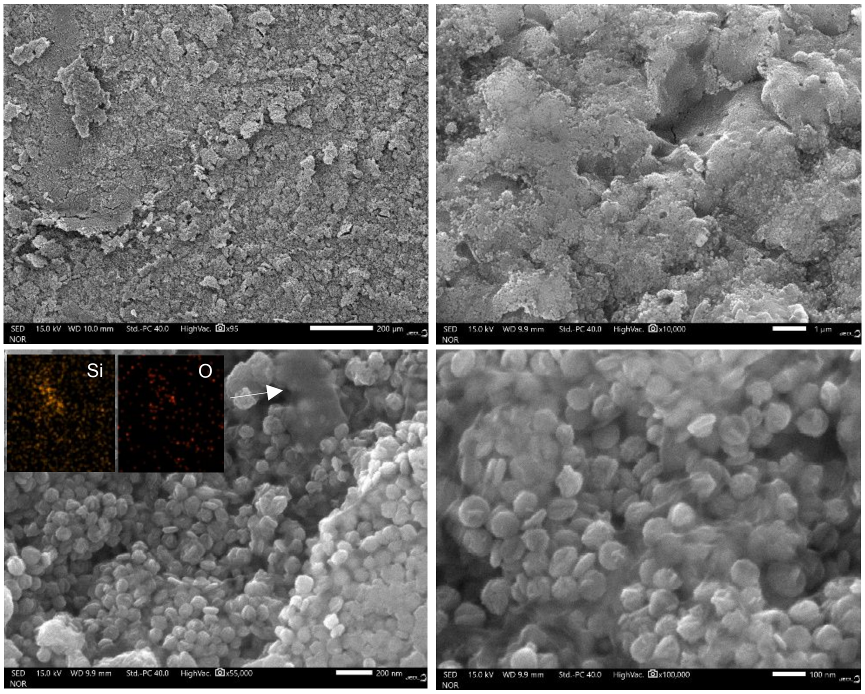


**Figure S13.** SEM images of $Cu_2S$ samples synthesized with 0.064 mL $N_2H_4$ (1x) at 10°C using $NH_3.H_2O$ as the base. As can be seen from the images, particles in this sample appear to be wrap around by some amorphous species. Further inspection by EDS elemental mapping of one such amorphous species (inset of the image at bottom-left corner) reveals that these species are likely to be $SiO_2$ dust that can have contaminated the sample during the imaging preparation process

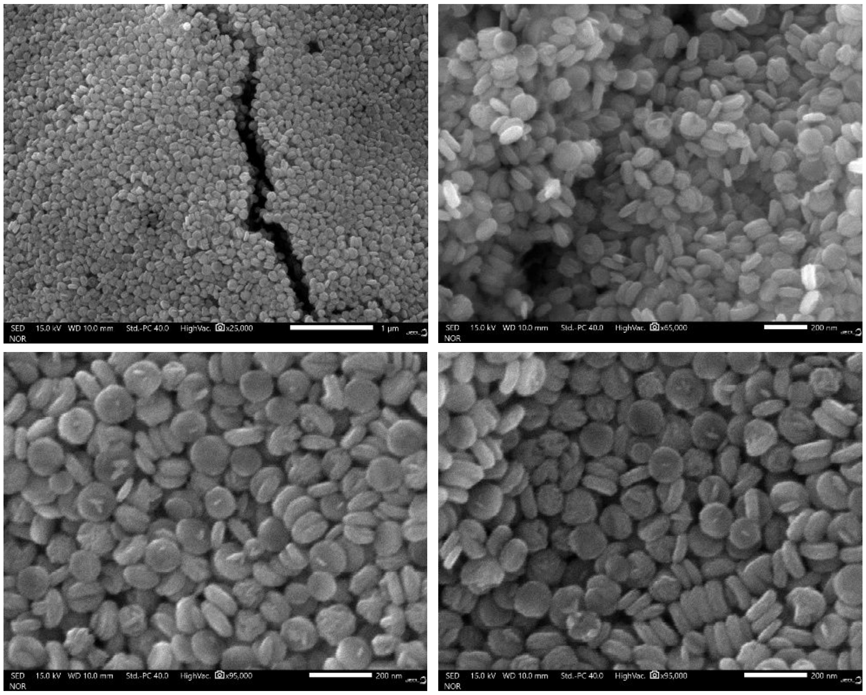

**Figure S14.** SEM images of $Cu_2S$ samples synthesized with 0.064 mL $N_2H_4$ (1x) at 20°C using $NH_3.H_2O$ as the base

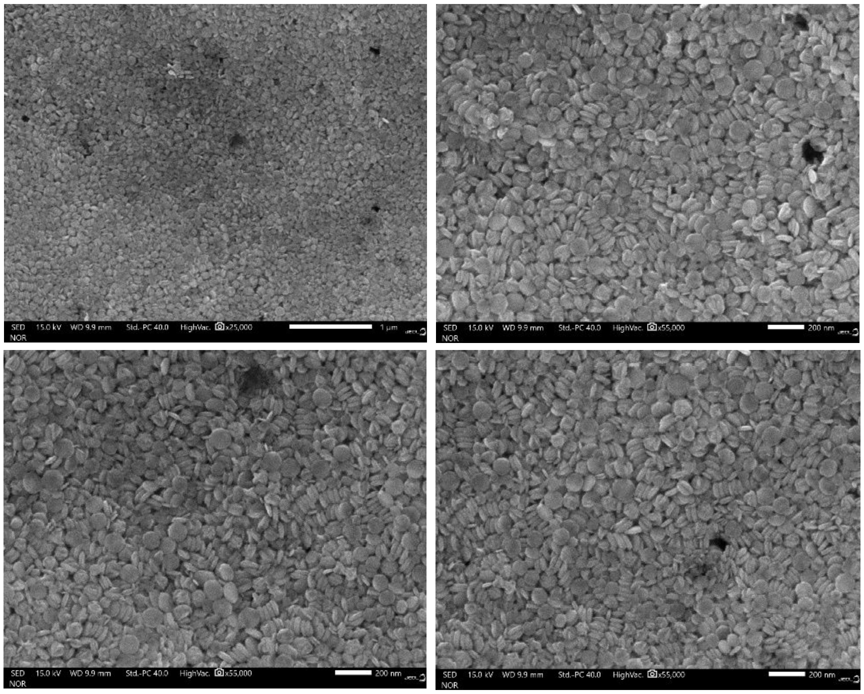

**Figure S15.** SEM images of $Cu_2S$ samples synthesized with 0.064 mL $N_2H_4$ (1x) at 30°C using $NH_3.H_2O$ as the base

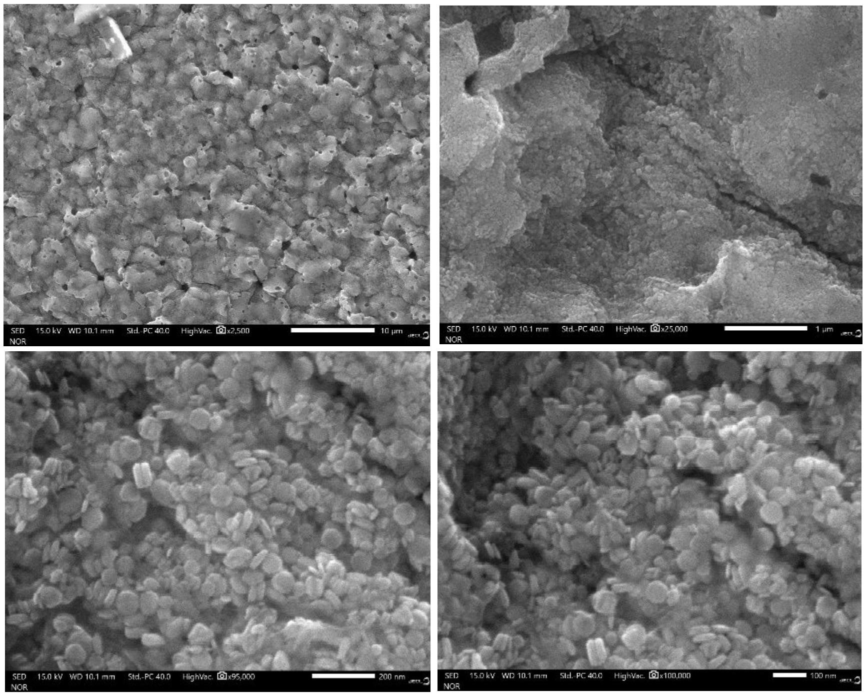

**Figure S16.** SEM images of $Cu_2S$ samples synthesized with 0.064 mL $N_2H_4$ (1x) at 40°C using $NH_3.H_2O$ as the base

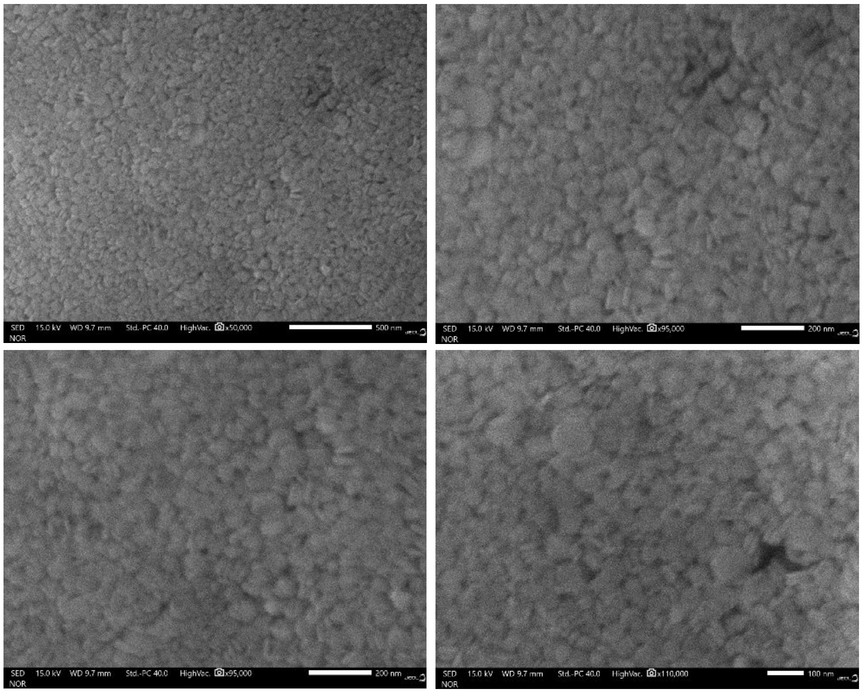

**Figure S17.** SEM images of $Cu_2S$ samples synthesized with 0.064 mL $N_2H_4$ (1x) at 50°C using $NH_3.H_2O$ as the base

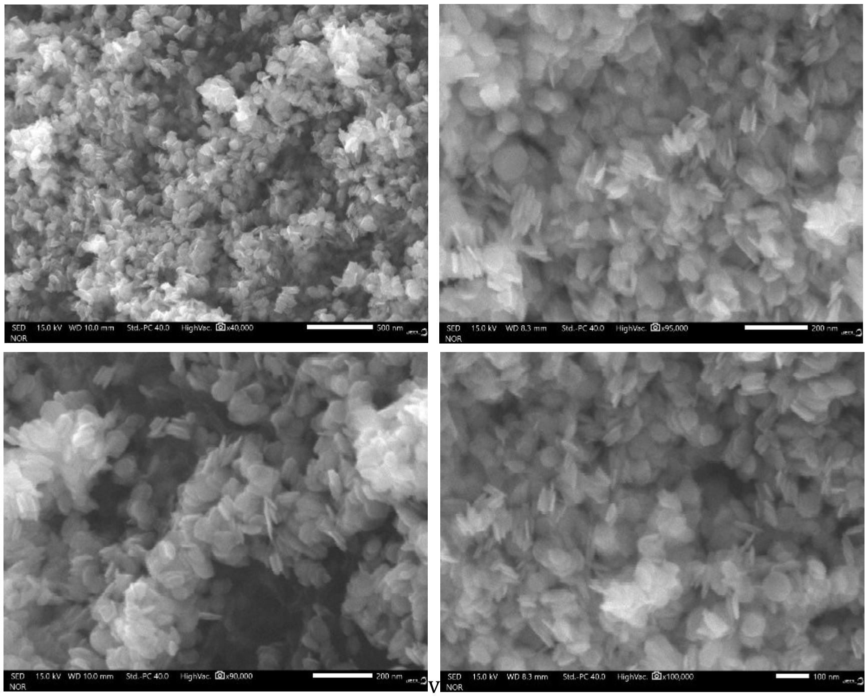

**Figure S18.** SEM images of $Cu_2S$ samples synthesized with 0.064 mL $N_2H_4$ (1x) at 60°C using $NH_3.H_2O$ as the base

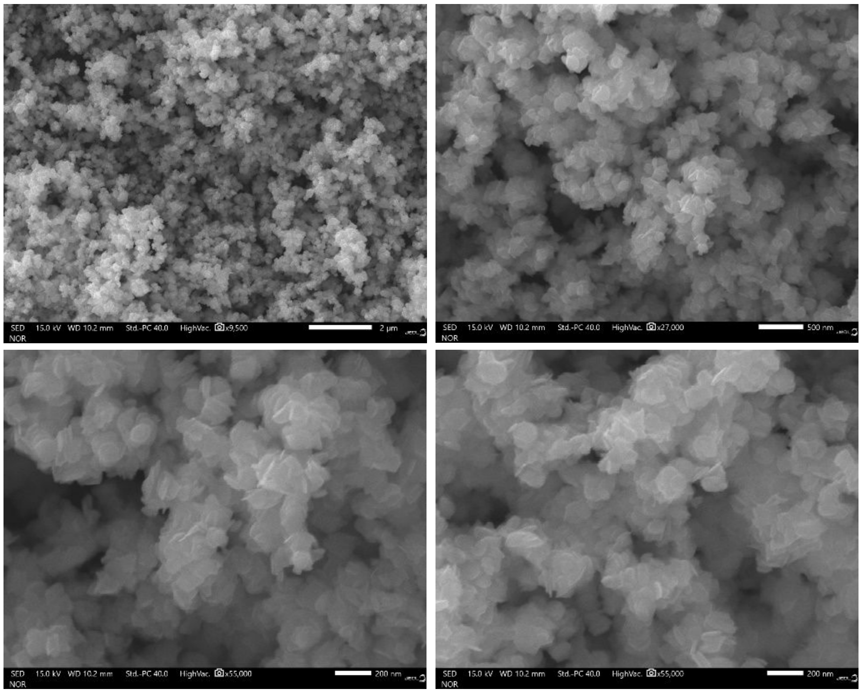

**Figure S19.** SEM images of $Cu_2S$ samples synthesized with 0.064 mL $N_2H_4$ (1x) at 70°C using $NH_3.H_2O$ as the base

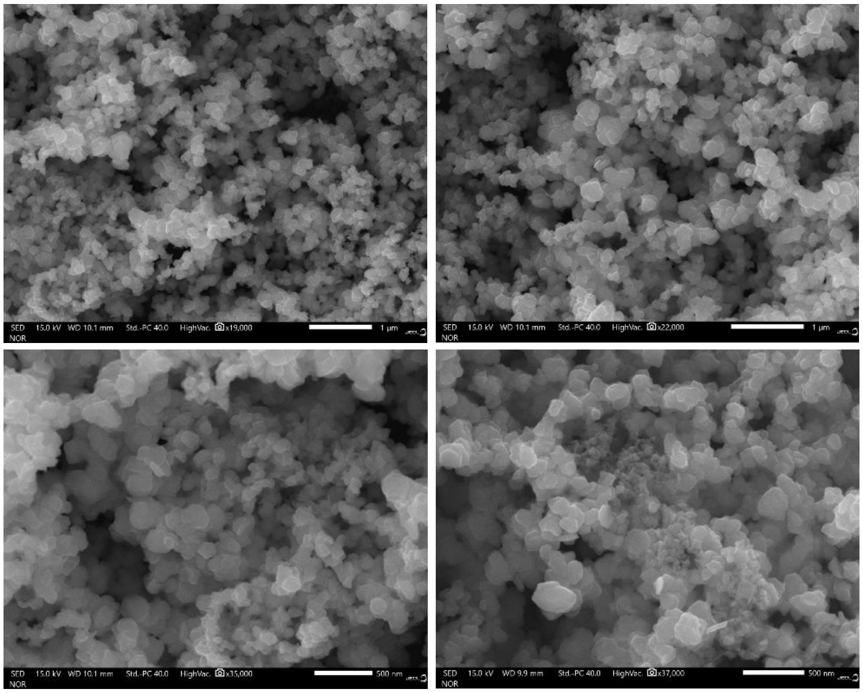

**Figure S20.** SEM images of $Cu_2S$ samples synthesized with 0.064 mL $N_2H_4$ (1x) at 80°C using $NH_3.H_2O$ as the base

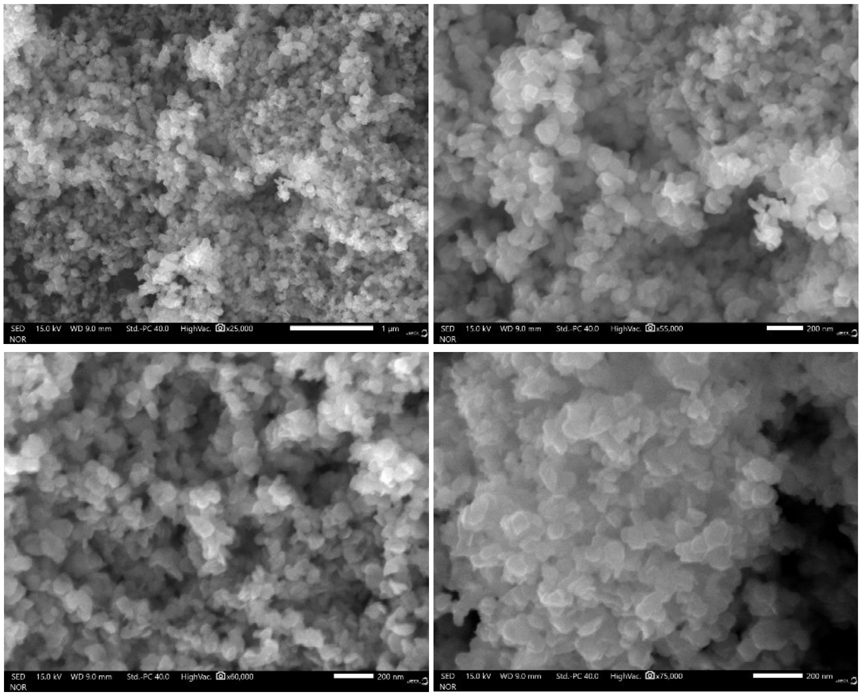

**Figure S21.** SEM images of $Cu_2S$ samples synthesized with 0.064 mL $N_2H_4$ (1x) at 90°C using $NH_3.H_2O$ as the base

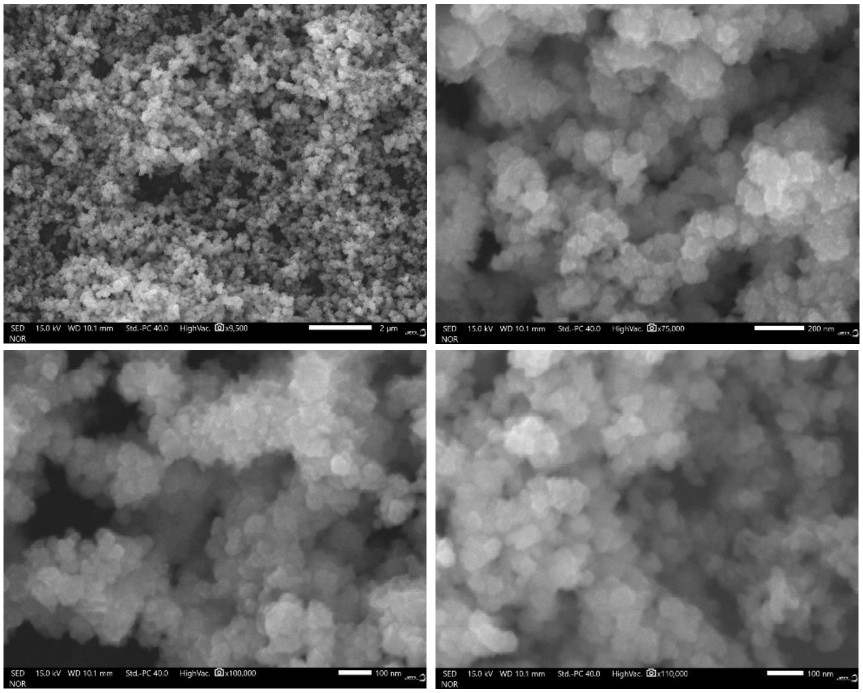

**Figure S22.** SEM images of $Cu_2S$ samples synthesized with 0.064 mL $N_2H_4$ (1x) at 100°C using $NH_3.H_2O$ as the base

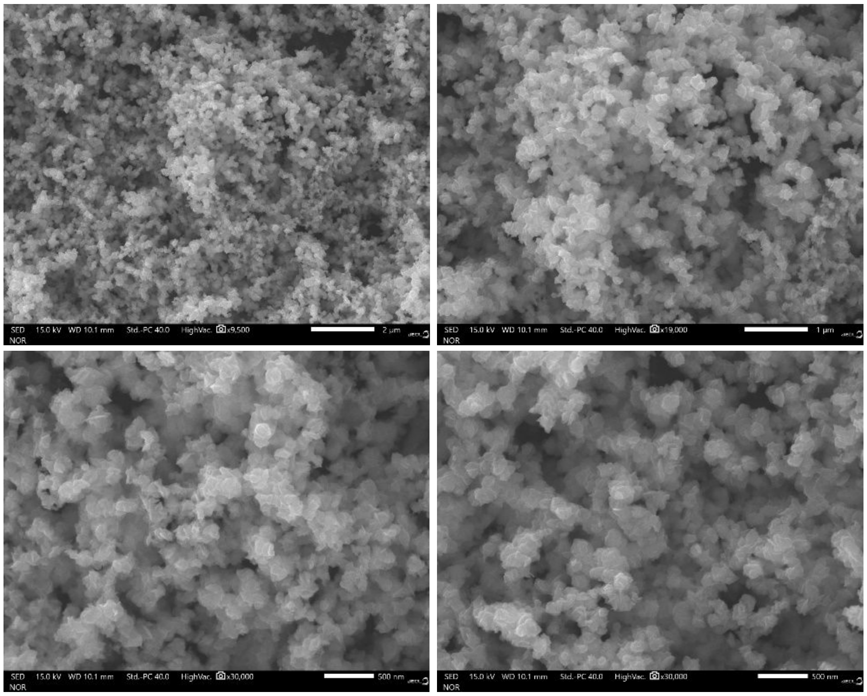

**Figure S23.** SEM images of $Cu_2S$ samples synthesized with 0.32 mL $N_2H_4$ (x5) at 40°C using NaOH as the base

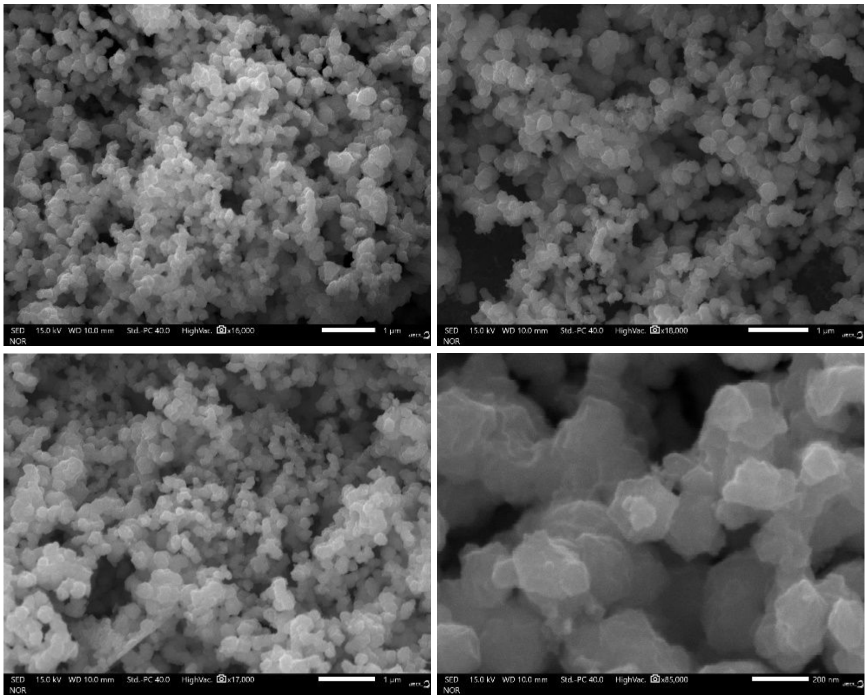

**Figure S24.** SEM images of $Cu_2S$ samples synthesized with 0.64 mL $N_2H_4$ (10x) at 40°C using NaOH as the base

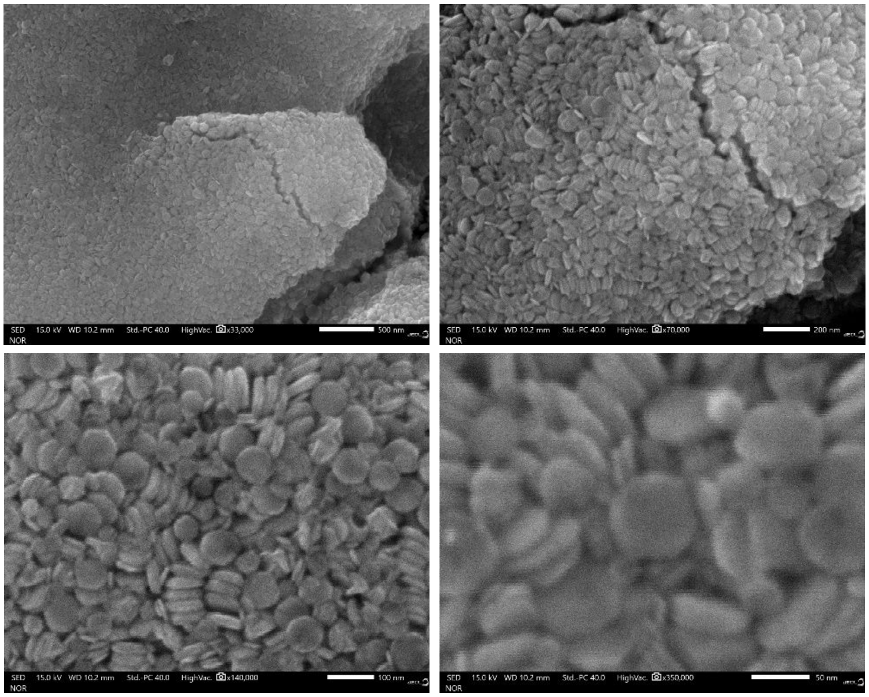

**Figure S25.** SEM images of $Cu_2S$ samples synthesized with 0.32 mL $N_2H_4$ (x5) at 40°C using $NH_3.H_2O$ as the base

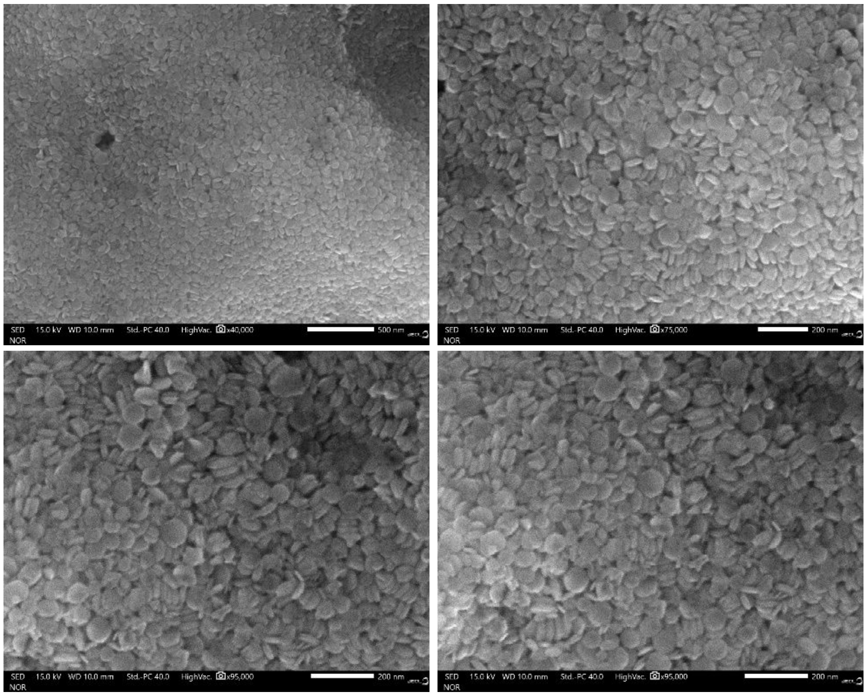

**Figure S26.** SEM images of $Cu_2S$ samples synthesized with 0.64 mL $N_2H_4$ (10x) at 40°C using $NH_3.H_2O$ as the base

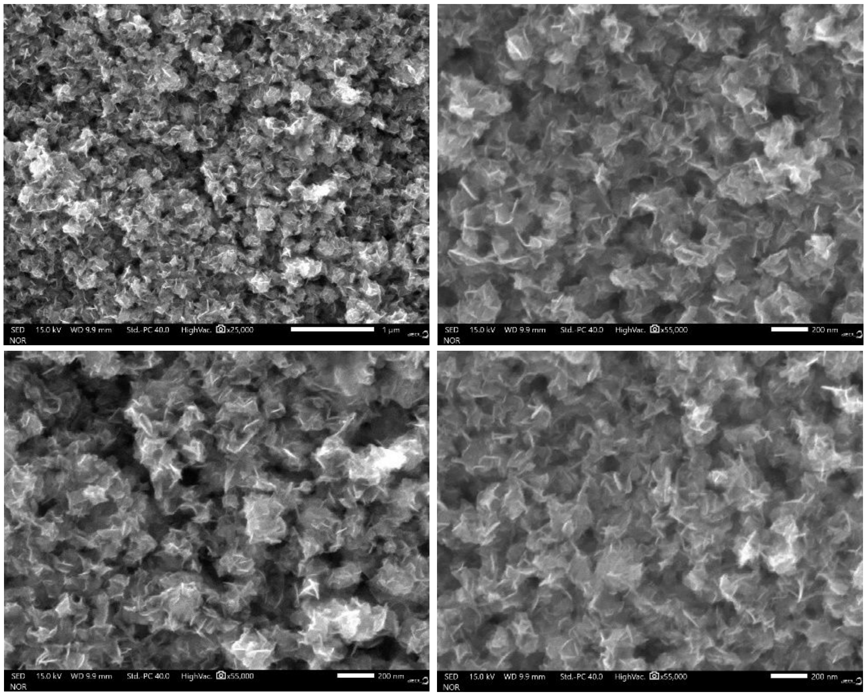

**Figure S27.** SEM images of $Cu_2S$ samples synthesized with 0.064 mL $N_2H_4$ (1x) at 25°C using NaOH as the base

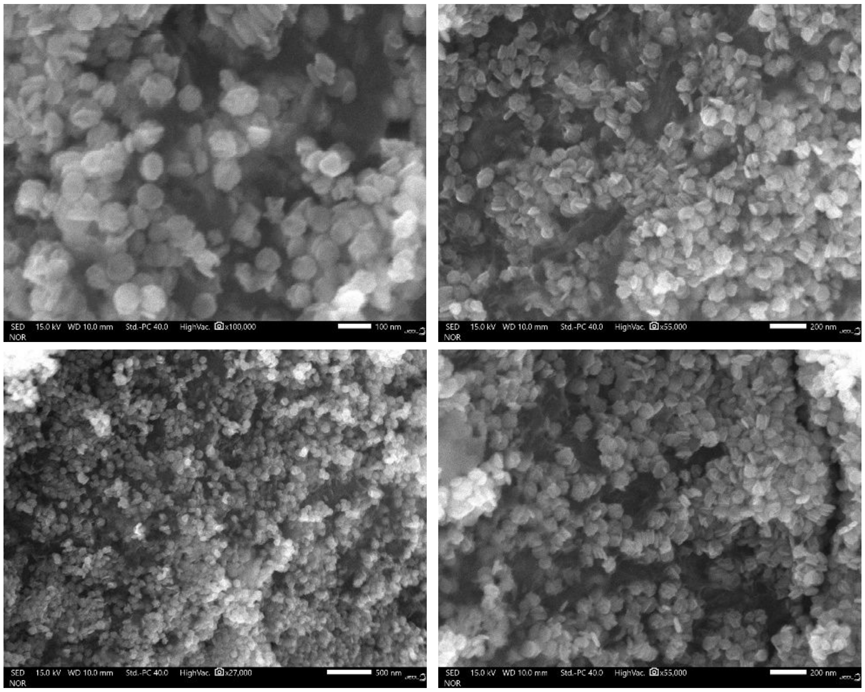

**Figure S28.** SEM images of $Cu_2S$ samples synthesized with 0.064 mL $N_2H_4$ (1x) at 25°C using $NH_3.H_2O$ as the base

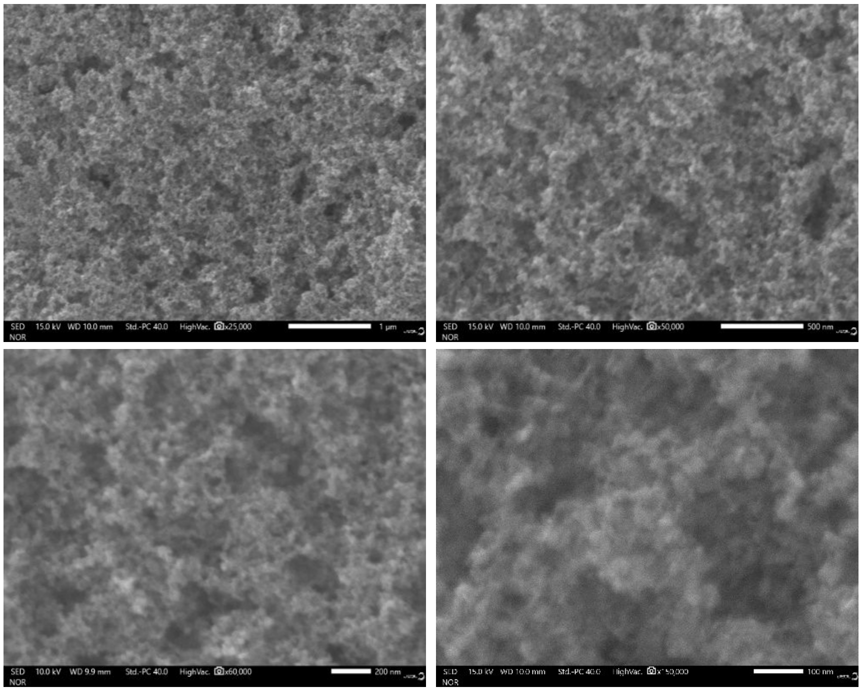

**Figure S29.** SEM images of $Cu_2S$ samples synthesized with 0.064 mL $N_2H_4$ (1x) at 25°C using $Et_3N$ as the base

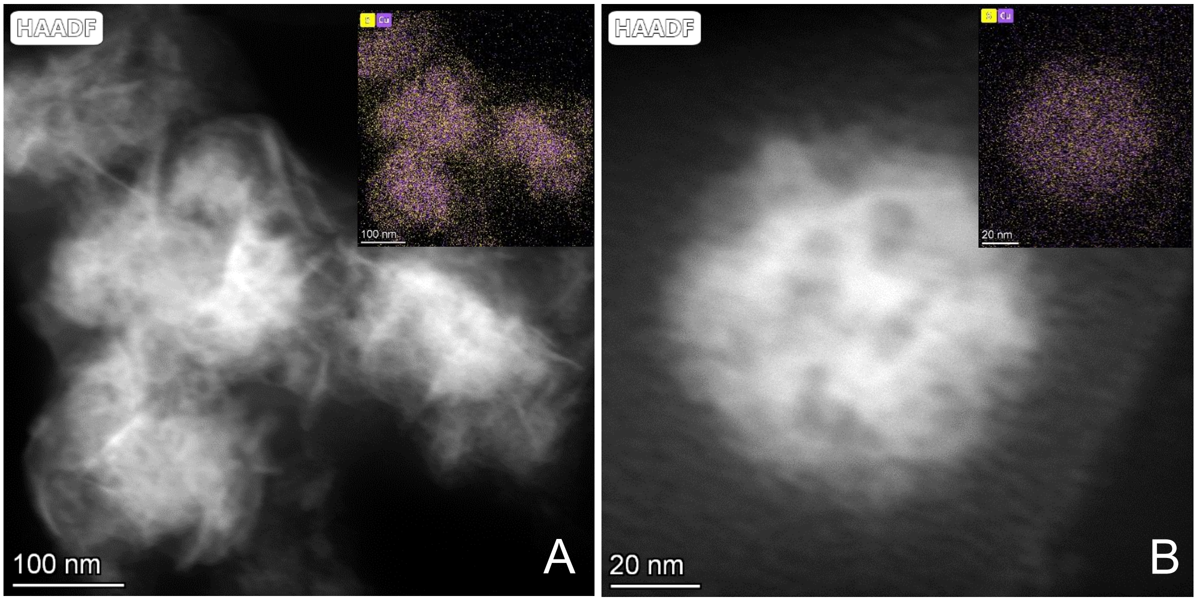


**Figure S30.** TEM images of $Cu_2S$ samples synthesized with 0.064 mL $N_2H_4$ (1x) at 25°C using (A) NaOH as the base and (b) $NH_3.H_2O$ as the base. On the inset are EDS elemental mapping of Cu and S

| Element | Line | Mass% | Atom% |
|---|---|---|---|
| O | K | 3.64 ± 0.21 | 10.99 ± 0.64 |
| S | K | 20.95 ± 0.39 | 31.60 ± 0.58 |
| Cu | K, L | 75.42 ± 2.16 | 57.41 ± 1.65 |
| Total | | 100.01 | 100.00 |

**Table S1.** EDS ZAF Quantification of $Cu_2S$ samples synthesized with 0.064 mL $N_2H_4$ (1x) at 25°C using NaOH as the base

| Element | Line | Mass% | Atom% |
|---|---|---|---|
| C | K | 11.03 ± 0.42 | 33.64 ± 1.29 |
| O | K | 3.24 ± 0.23 | 7.33 ± 0.53 |
| S | K | 16.93 ± 0.38 | 19.34 ± 0.43 |
| Cu | K, L | 68.84 ± 2.28 | 39.69 ± 1.31 |
| Total | | 100.0 | 100.00 |

**Table S2.** EDS ZAF Quantification of $Cu_2S$ samples synthesized with 0.064 mL $N_2H_4$ (1x) at 25°C using $NH_3.H_2O$ as the base. Carbon can be spotted which is likely due to the usage of carbon paint for the imaging preparation process

| Element | Line | Mass% | Atom% |
|---|---|---|---|
| C | K | 6.58 ± 0.10 | 22.92 ± 0.35 |
| O | K | 1.23 ± 0.04 | 3.21 ± 0.11 |
| Si | K | 0.31 ±0.03 | 0.46 ± 0.04 |
| S | K | 19.99 ± 0.12 | 26.08 ± 0.16 |
| Cu | K, L | 71.90 ± 0.69 | 47.33 ± 0.46 |
| Total | | 100.0 | 100.00 |

**Table S3.** EDS ZAF Quantification of $Cu_2S$ samples synthesized with 0.064 mL $N_2H_4$ (1x) at 25°C using $Et_3N$ as the base. Carbon can be spotted which is likely due to the usage of carbon paint for the imaging preparation process whereas silicon can likely be attributed to dust contamination

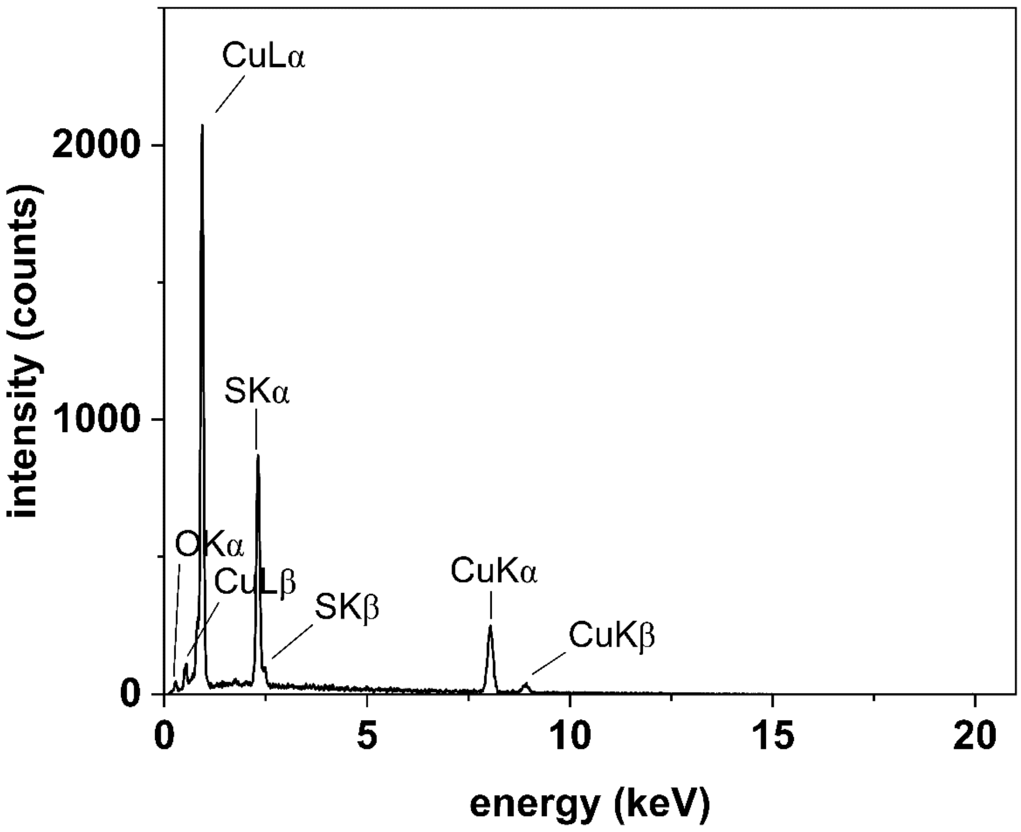


**Figure S31.** EDS elemental mapping of $Cu_2S$ samples synthesized with 0.064 mL $N_2H_4$ (1x) at 25°C using NaOH as the base

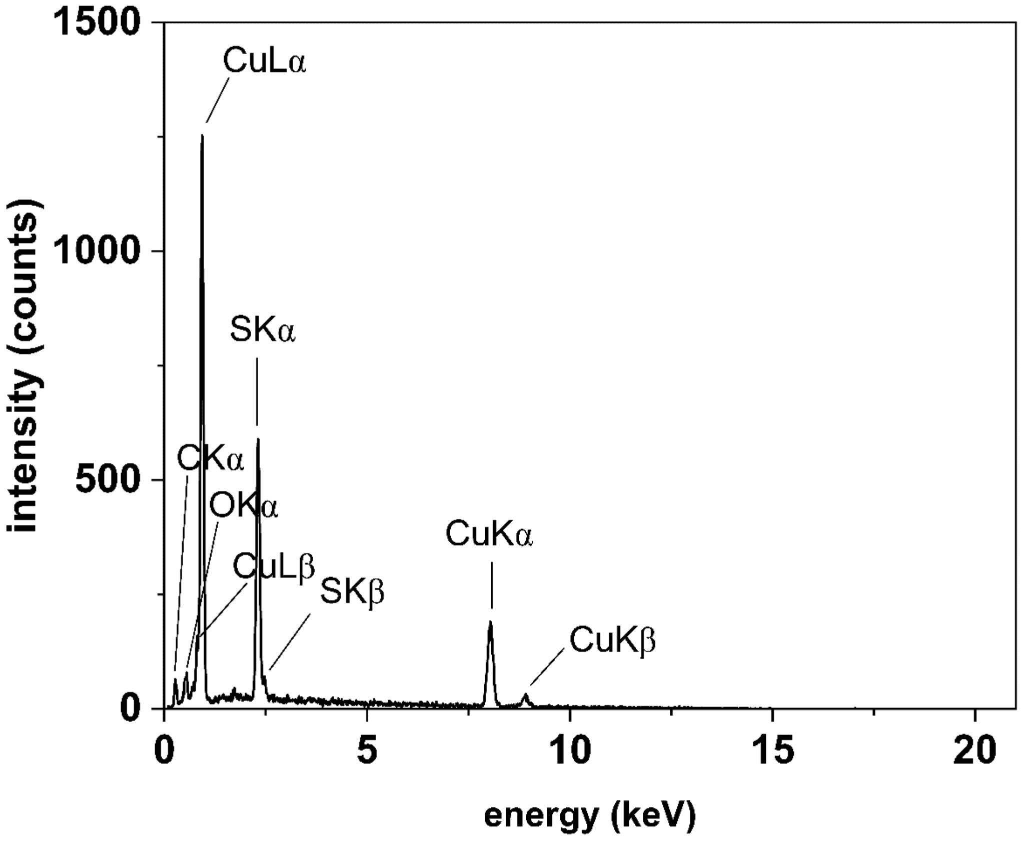


**Figure S32.** EDS elemental mapping of $Cu_2S$ samples synthesized with 0.064 mL $N_2H_4$ (1x) at 25°C using $NH_3.H_2O$ as the base

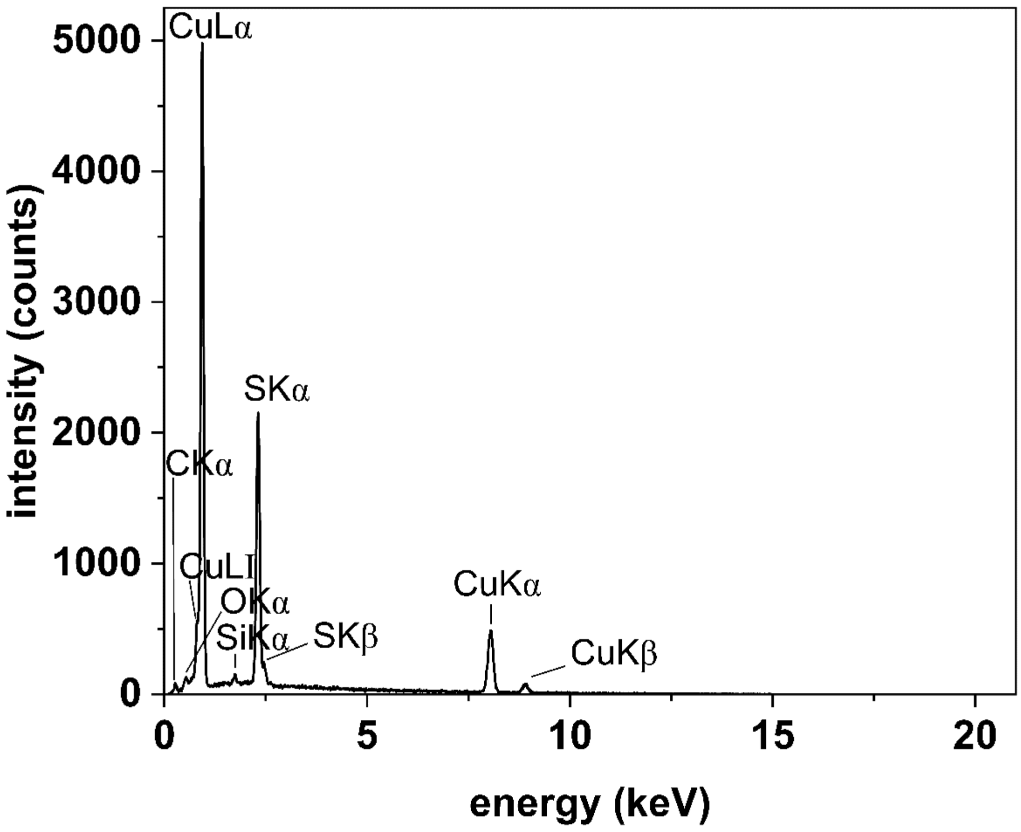


**Figure S33.** EDS elemental mapping of $Cu_2S$ samples synthesized with 0.064 mL $N_2H_4$ (1x) at 25°C using $Et_3N$ as the base

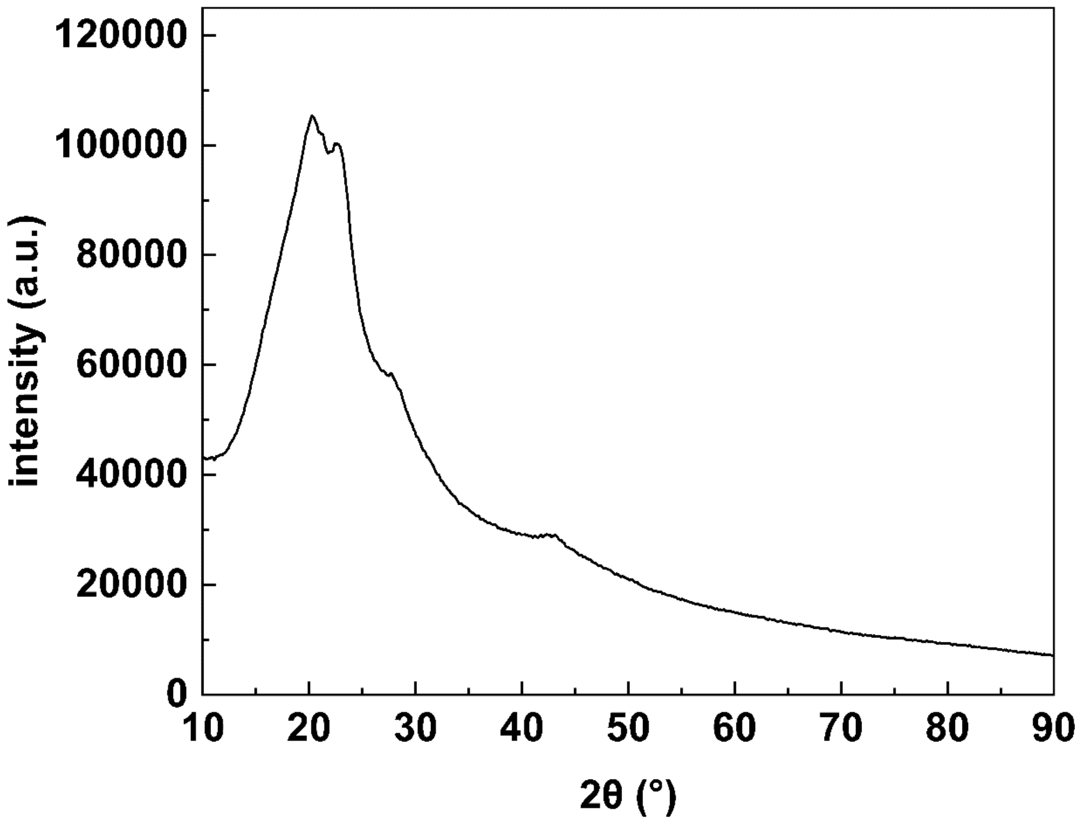


**Figure S34.** XRD graph of 2% aramid nanofibers

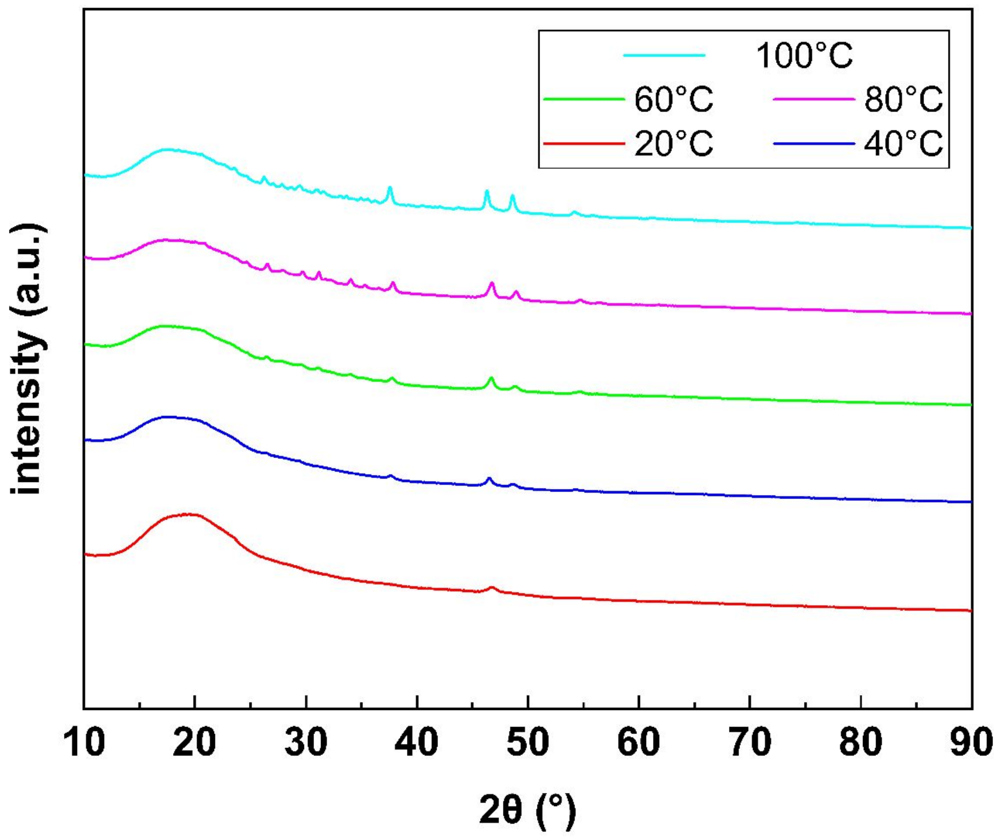


**Figure S35.** XRD graph of $Cu_2S$ samples synthesized with 0.064 mL $N_2H_4$ (1x) using NaOH as the base at different temperatures

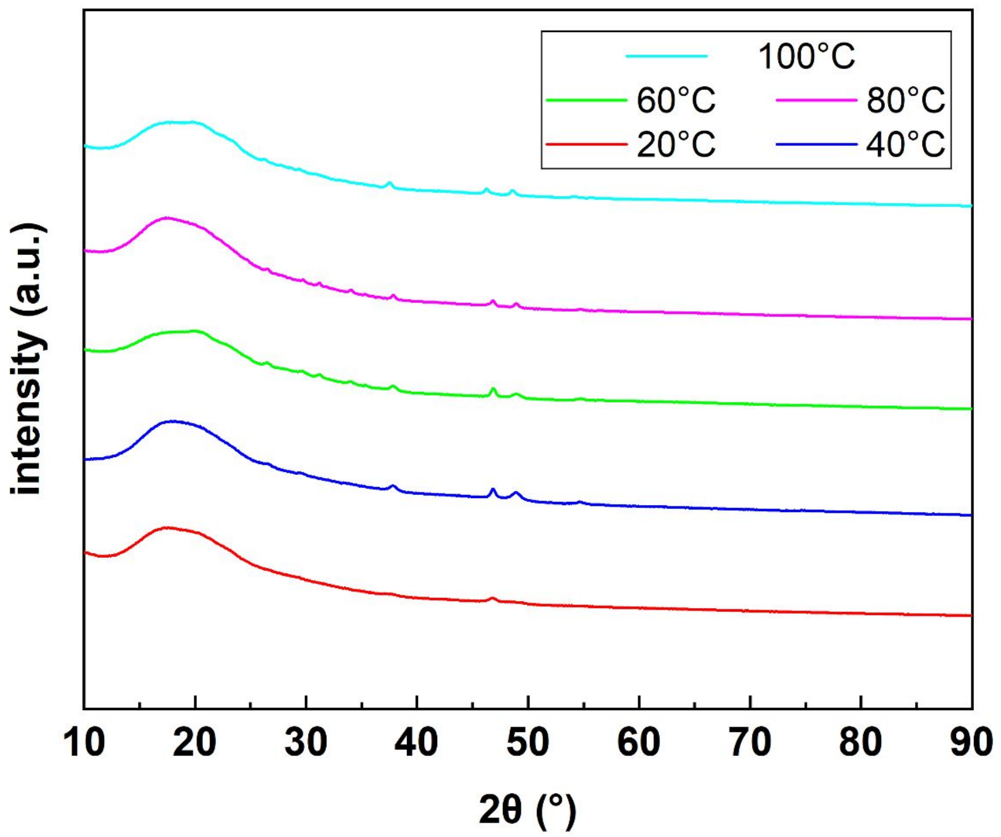


**Figure S36.** XRD graph of $Cu_2S$ samples synthesized with 0.064 mL $N_2H_4$ (1x) using $NH_3.H_2O$ as the base at different temperatures

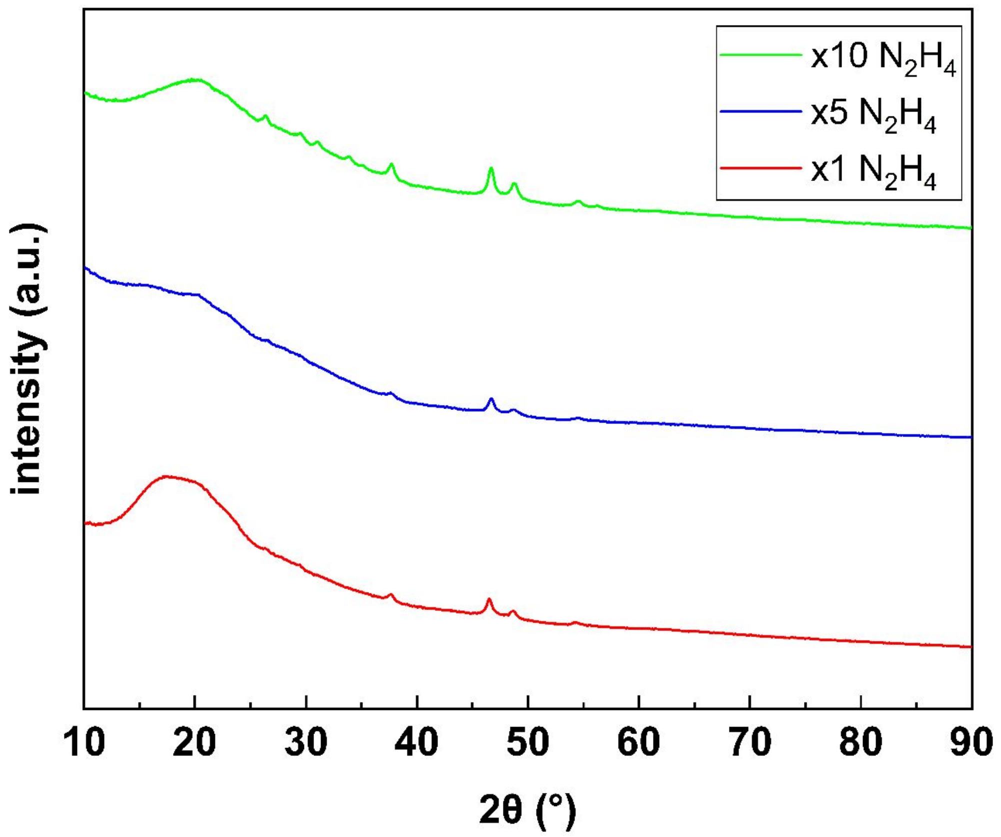


**Figure S37.** XRD graph of $Cu_2S$ samples synthesized with different concentrations of $N_2H_4$ at 25°C using NaOH as the base

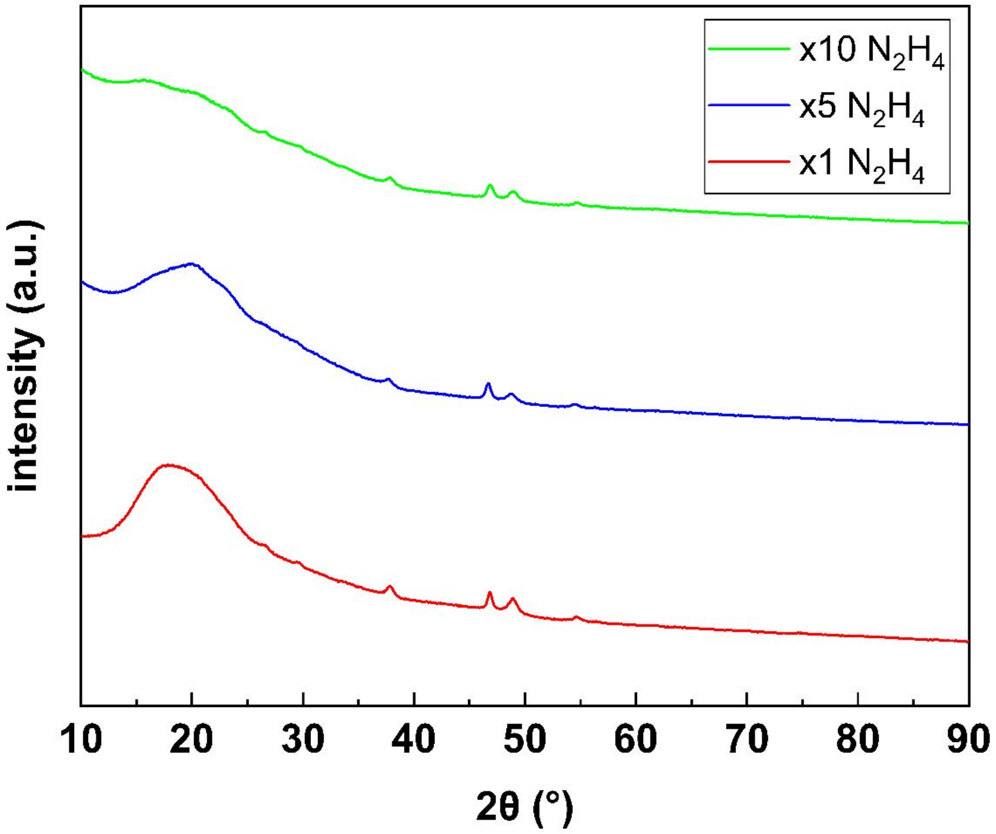


**Figure S38.** XRD graph of $Cu_2S$ samples synthesized different concentrations of $N_2H_4$ at 25°C using $NH_3.H_2O$ as the base

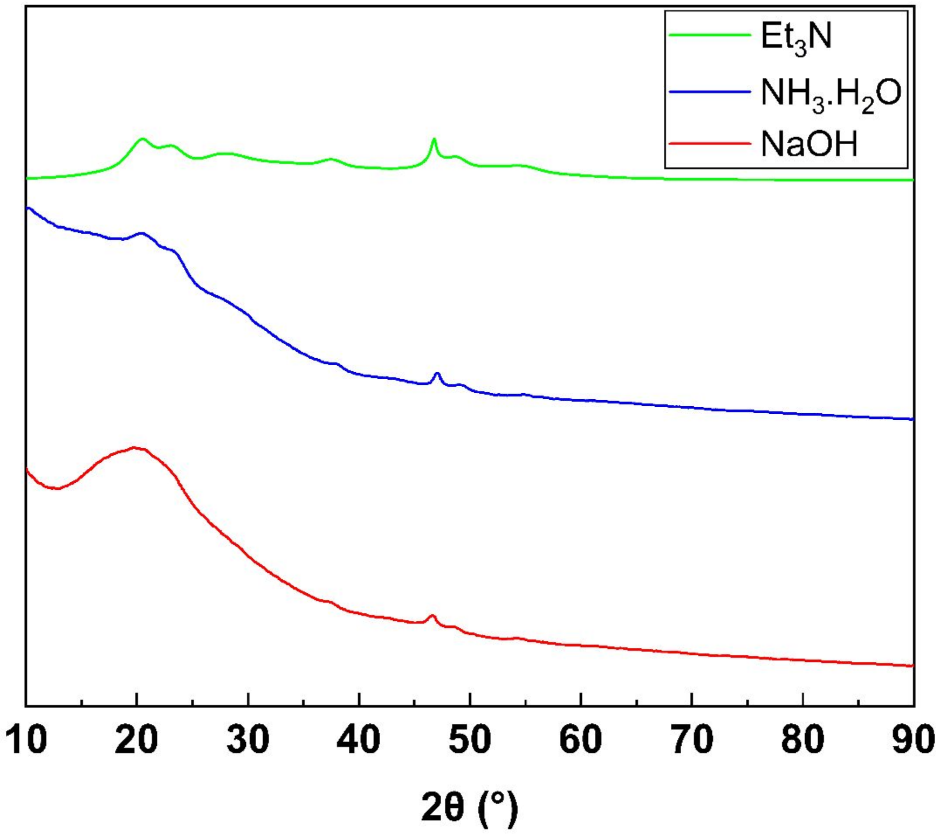


**Figure S39.** XRD graph comparing $Cu_2S$ samples synthesized with 0.064 mL $N_2H_4$ at 25°C using NaOH, $NH_3.H_2O$, and $Et_3N$

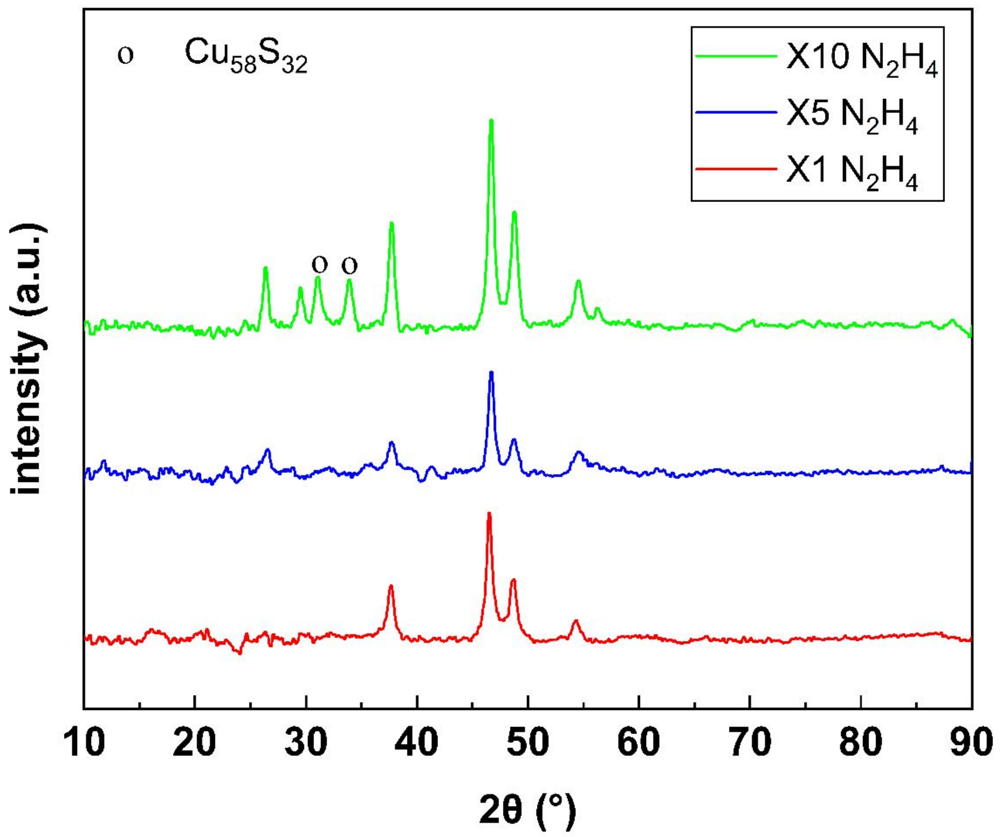


**Figure S40.** Fitted-XRD graph of $Cu_2S$ samples synthesized with different concentrations of $N_2H_4$ at 25°C using NaOH as the base. Except from the peaks at around 30.96° (1 -3 -4) and 33.54° (5 1 0) that are annotated with the letter O which are likely to belong to the trace amount of $Cu_{58}S_{32}$ roxbyite (00-064-0278 : PDF-5+ 2024), the rest of the peaks are indicative of $Cu_2S$ chalcocite (04-010-5153 : PDF-5+ 2024)

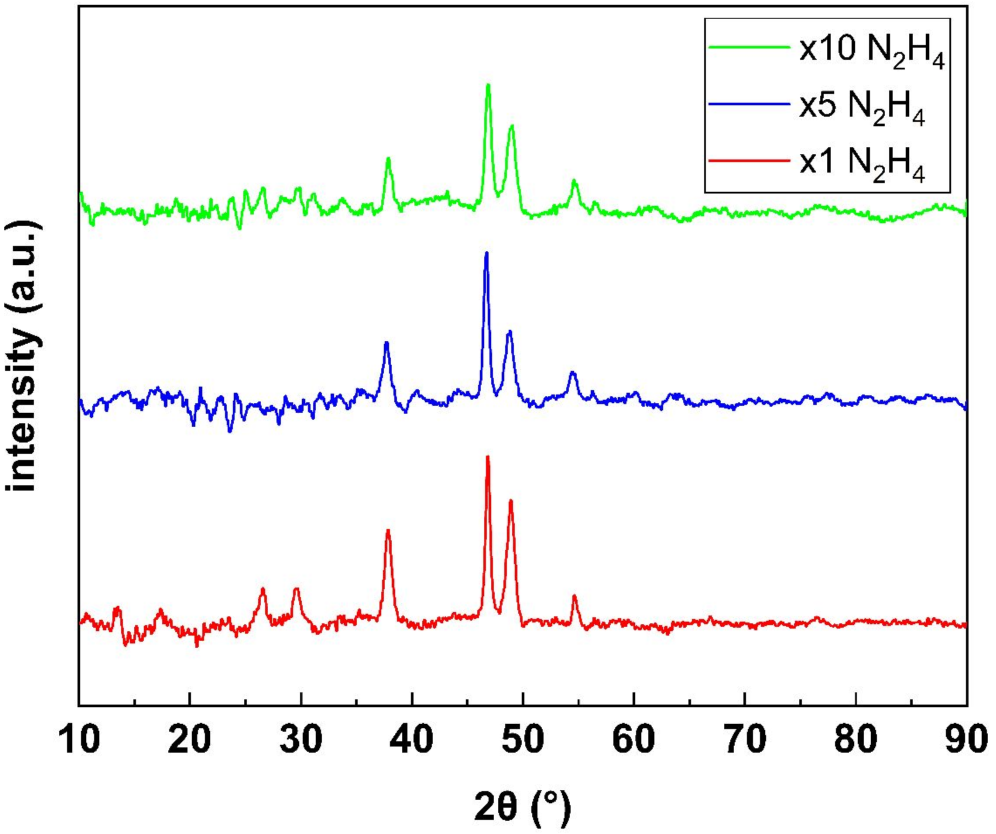


**Figure S41.** Fitted-XRD graph of $Cu_2S$ samples synthesized with different concentration of $N_2H_4$ at 25°C using $NH_3.H_2O$ as the base. All the peaks are indicative of $Cu_2S$ chalcocite (04-010-5153 : PDF-5+ 2024)

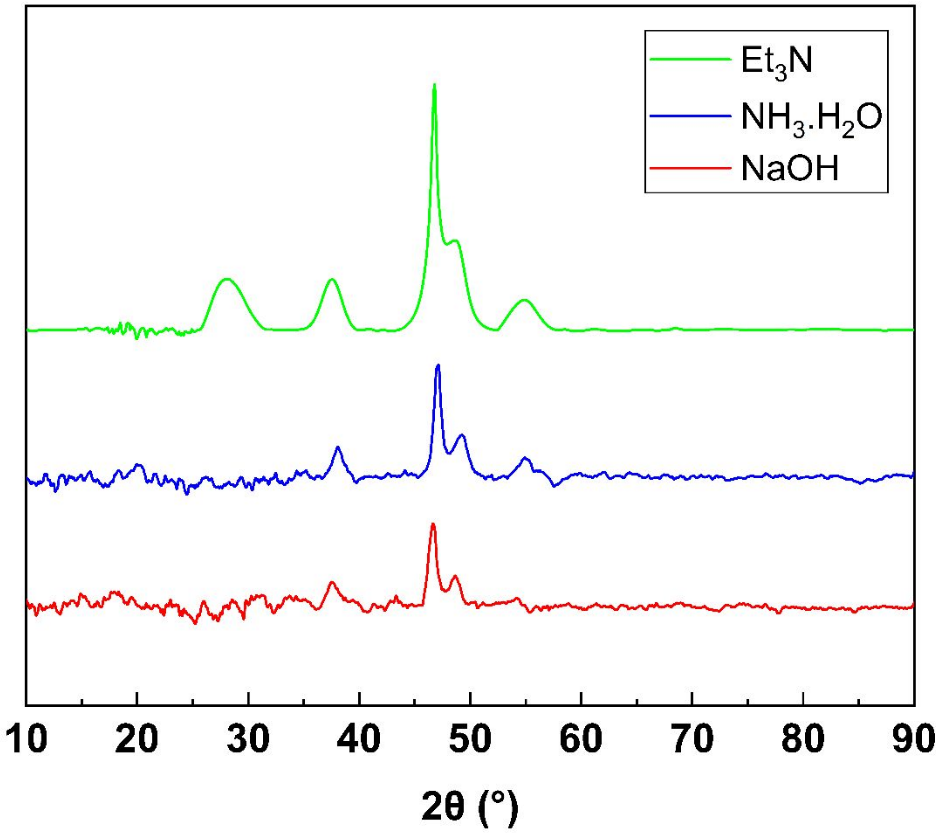


**Figure S42.** Fitted-XRD graph comparing $Cu_2S$ samples synthesized with 0.064 mL $N_2H_4$ (1x) at 25°C using NaOH, $NH_3.H_2O$, and $Et_3N$. All the peaks are indicative of $Cu_2S$ chalcocite (04-010-5153 : PDF-5+ 2024)

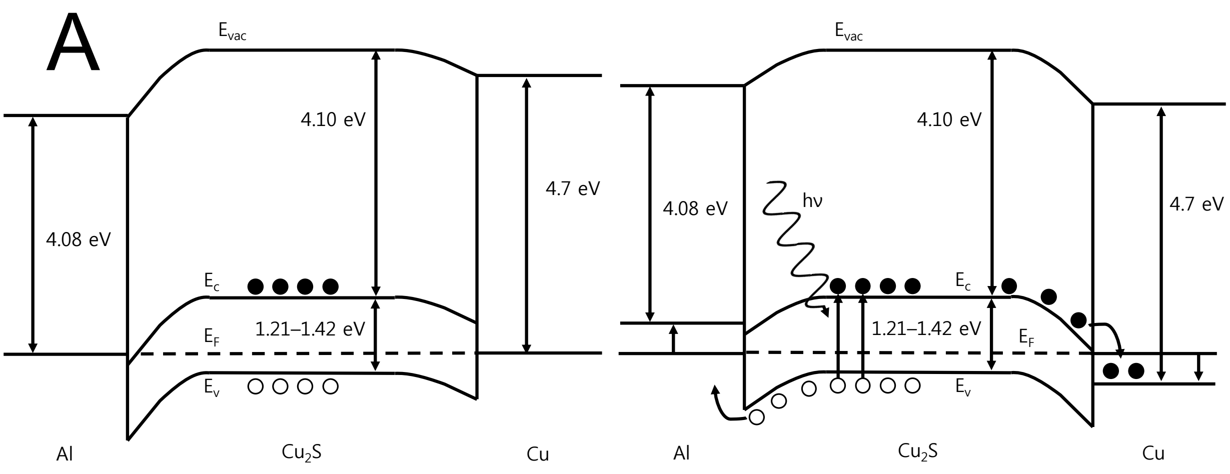


**Figure S43.** Energy band diagram of the sample (A) without and (B) with high voltage. Band gap of $Cu_2S$ was measured in **Section 3.6**; Work functions of the metals[1] and electron affinity of $Cu_2S$[2] were referred from previous studies.

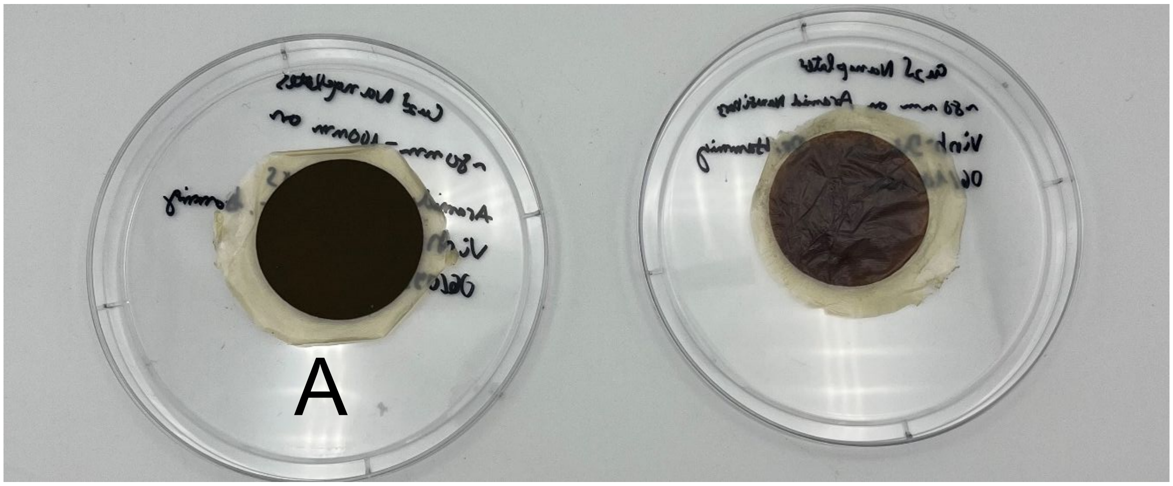


**Figure S44.** $Cu_2S$/Aramid Nanofiber composites from $Cu_2S$ synthesized with 0.064 mL $N_2H_4$ at 25°C using (A) NaOH and (B) $NH_3.H_2O$. Note that the exterior of the aramid nanofibers that is not integrated with $Cu_2S$ nanoplates has been cut off before the volume density calculation is conducted

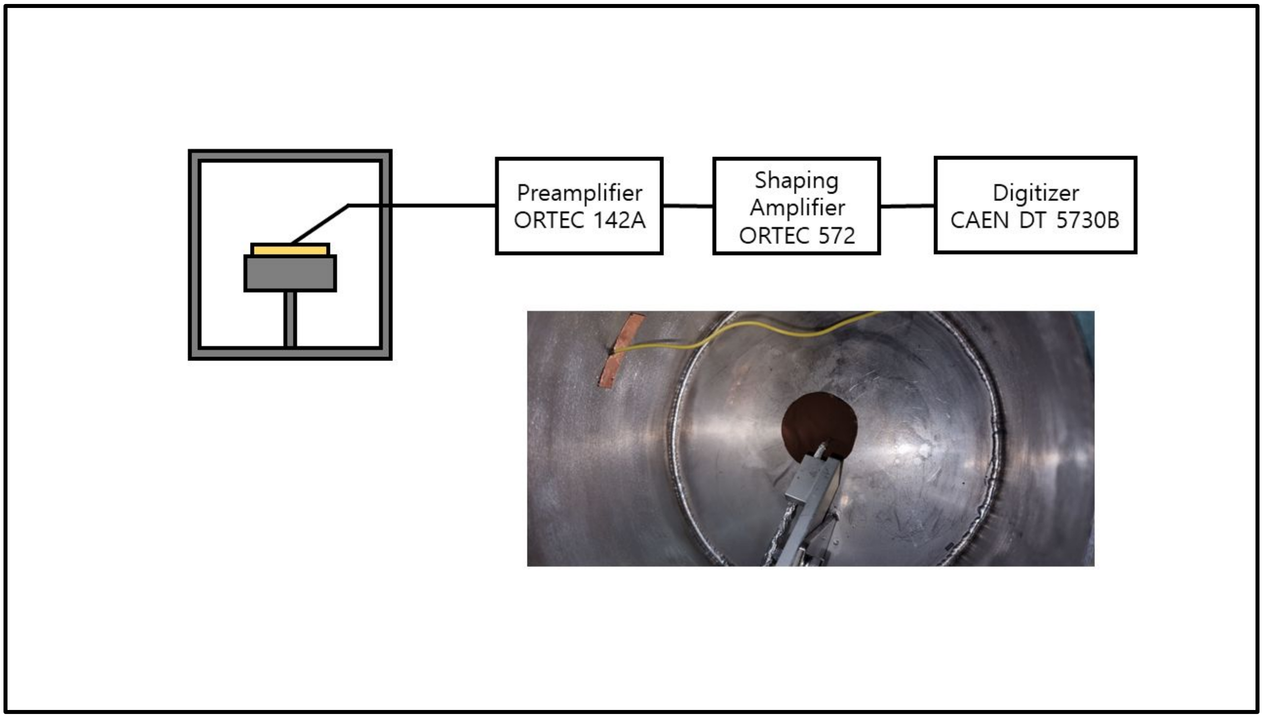


**Figure S45.** Experimental setup of photonic response measurement for $Cu_2S$/Aramid Nanofiber composites from $Cu_2S$ synthesized with 0.064 mL $N_2H_4$ at 25°C using NaOH

## References


(1) Stössel, M.; Staudigel, J.; Steuber, F.; Simmerer, J.; Winnacker, A. Impact of the Cathode Metal Work Function on the Performance of Vacuum-Deposited Organic Light Emitting-Devices. *Appl Phys A* **1999**, *68* (4), 387–390. https://doi.org/10.1007/s003390050910.

(2) Lima, J. V. M.; Santos, S. B. O.; Silva, R. A.; Boratto, M. H.; Graeff, C. F. O.; Scalvi, L. V. A. Anomalous Diode Behavior of Cu2S/SnO2 p–n Junction. *J Mater Sci: Mater Electron* **2021**, *32* (16), 21804–21812. https://doi.org/10.1007/s10854-021-06703-x.
